# Nonlinear optimal control of a mean-field model of neural population dynamics

**DOI:** 10.3389/fncom.2022.931121

**Published:** 2022-08-03

**Authors:** Lena Salfenmoser, Klaus Obermayer

**Affiliations:** Institute of Software Engineering and Theoretical Computer Science, Technical University of Berlin, Berlin, Germany

**Keywords:** nonlinear optimal control, control of neural dynamics, neural mass models, bistability, delay differential-algebraic equations (DDAEs), nonlinear population dynamics

## Abstract

We apply the framework of nonlinear optimal control to a biophysically realistic neural mass model, which consists of two mutually coupled populations of deterministic excitatory and inhibitory neurons. External control signals are realized by time-dependent inputs to both populations. Optimality is defined by two alternative cost functions that trade the deviation of the controlled variable from its target value against the “strength” of the control, which is quantified by the integrated 1- and 2-norms of the control signal. We focus on a bistable region in state space where one low- (“down state”) and one high-activity (“up state”) stable fixed points coexist. With methods of nonlinear optimal control, we search for the most cost-efficient control function to switch between both activity states. For a broad range of parameters, we find that cost-efficient control strategies consist of a pulse of finite duration to push the state variables only minimally into the basin of attraction of the target state. This strategy only breaks down once we impose time constraints that force the system to switch on a time scale comparable to the duration of the control pulse. Penalizing control strength *via* the integrated 1-norm (2-norm) yields control inputs targeting one or both populations. However, whether control inputs to the excitatory or the inhibitory population dominate, depends on the location in state space relative to the bifurcation lines. Our study highlights the applicability of nonlinear optimal control to understand neuronal processing under constraints better.

## 1. Introduction

Optimal control theory (OCT) provides a toolbox to investigate the effect of targeted perturbations on dynamical systems (Berkovitz and Medhin, 2012). It enables to answer the question of how stimulation must be designed to optimally induce or stop specific dynamical states or activity patterns. Optimality is defined through the global minimum of a cost function, which typically rewards closeness to desired target values of the state variables and penalizes control effort, which can be quantified, for example, in terms of the duration and strength of an external control signal (Casas et al., 2015). Applications of OCT are 2-fold. In a “synthetic” application scenario, OCT can help us to manipulate a dynamical system optimally, for example, to follow the desired trajectory. In an “analytic” application scenario, it can help us understand the way in which a natural dynamical system is designed and offers explanations of its workings in terms of optimization principles. In the past, OCT has been applied successfully in biology and biomedicine with applications to cellular systems, metabolic networks, and the development of effective treatments against pathogens (see, e.g., Ewald et al., 2017; Tsiantis and Banga, 2020 for recent reviews).

Applications to neural systems have been mostly on the synthetic side so far and cover a variety of open and closed loop approaches for modulating brain activity (cf. Grosenick et al., 2015; Tafazoli et al., 2020; Takeuchi and Berényi, 2020). Examples include deep brain stimulation for the treatment of patients with Parkinson's disease (Popovych and Tass, 2019), invasive stimulation to imprint population activity (Marshel et al., 2019), e.g., in the context of neuro-prosthetic devices (Chen et al., 2020; Flesher et al., 2021), and non-invasive transcranial electrical stimulation for modulating and improving perception, motor control, and cognition (Au et al., 2017; Colzato et al., 2017; Reteig et al., 2017). Applications of OCT, however, are few and are mostly restricted to theoretical investigations. OCT in form of minimum-energy or minimum-power control strategies was applied to phase oscillators, which were derived to match single neuron phase response curves (Nabi et al., 2012; Dasanayake and Li, 2014; Pyragas et al., 2020). Here, the first experimental verifications of this technique confirmed an improved performance (Wilson et al., 2015). OCT was applied more extensively to wave propagation in systems of coupled non-linear oscillators (cf. Löber and Engel, 2014; Ziepke et al., 2019; Shangerganesh and Sowndarrajan, 2020), which also serve as models for neurons or neural populations, but closer links to the neuroscience literature were not yet made.

Compared to other applications in biology and biomedicine, there have been fewer works exploring the potential of OCT for analytic investigations into neural systems. One exception is motor control, for which OCT and optimal feedback control theory are well-established frameworks and drive theoretical analysis and modeling on a behavioral level (Todorov and Jordan, 2002; Diedrichsen et al., 2009; Scott, 2012). Beyond validating its applicability (Bian et al., 2020), recent studies extend this framework by including feedforward strategies (Yeo et al., 2016) and stochastic effects (Berret et al., 2021). Studies on applications of OCT to neural dynamics are few. Bassett and colleagues (cf. Gu et al., 2015; Tang and Bassett, 2018; Srivastava et al., 2020) applied diagnostics from linear control theory to the dynamics of neural populations in a whole-brain network setting, arguing that linearization is a valid approximation locally. Questions that were addressed include the efficacy of network nodes to steer the network dynamics, with some of the obtained results being confirmed by numerical simulations of a corresponding non-linear model (Muldoon et al., 2016). Results were interpreted in the context of the brain's internal control of general neurophysiological processes with implications for brain development and cognitive function, but also in the context of controlling altered neurophysiological processes in a medical context. A recent work (Ref. Chouzouris et al., 2021) applied nonlinear OCT to a whole-brain network model of FitzHugh-Nagumo oscillators, discussing the predictions of linear control diagnostics vs. nonlinear optimal control for different control settings. These studies highlight the potential of control theoretic concepts in an “analytic” setting for a mechanistic understanding of neural dynamics.

In this contribution, we explore the potential of OCT for predicting optimal perturbations for a motif, which consists of two recurrently connected populations of excitatory and inhibitory neurons and which is a common building block of many neural systems. We consider a biophysically grounded two population mass model (Cakan and Obermayer, 2020), whose populations are mathematically described *via* mean-field approximations of infinitely large populations of exponential integrate-and-fire (EIF) neurons (Brette and Gerstner, 2005; Augustin et al., 2017) and which exhibits down-states, up-states, and several oscillatory phenomena observed in neural systems. Here, we focus on a region in state space, in which the model is bistable, i.e., in which stable states of constant high and low activity coexist. We then apply nonlinear OCT in search of the most efficient strategies (in terms of accuracy and required control strength) for an external input to steer the motif from one of its stable fixed points to the other. To do so, we implement a gradient descent algorithm minimizing a cost function, which trades accuracy (w.r.t. the control goal) against control strength (measured by the integrated 1- and 2-norms of the control signal). We first explore the performance of the optimization method and explore its limitations. When applied to the switching task we find that—in the noiseless case—low-cost control strategies exploit the intrinsic properties of the dynamical system by steering the system just slightly across the boundary to the target attractor, from where the system converges to its target state without further external input. We then apply the OCT ansatz to inquire whether it is more efficient to steer the system *via* inputs to the inhibitory or the excitatory population if control strength is constrained. Penalizing control strength *via* the integrated 1-norm we find that the answer depends on the exact location of the system in state space. Thus, optimal control may require changing control inputs between the participating neural populations when the dynamical context is changed. These results show that OCT is a valuable tool and highlight its applicability to probe the dynamics of a nonlinear neural system.

This work is structured as follows. Section 2 introduces the mean-field model and its dynamics, formalizes the optimal control problem mathematically, and finally describes the numerical implementation of our optimal control algorithm. In Section 3, we explain the setup for the experiments and present our main findings. Section 4 concludes with a brief discussion and comments on the potential and shortcomings of our approach.

## 2. Methods

### 2.1. The neural mass model

**Figure 1:**
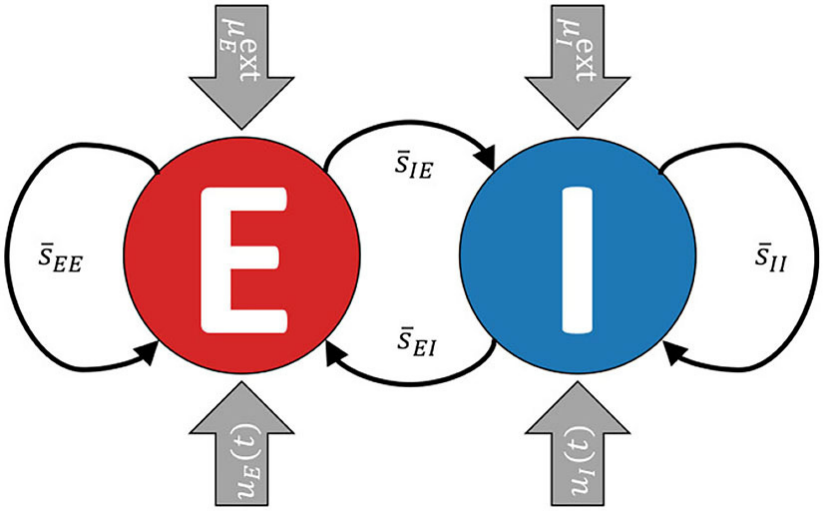
A simplified visualization of the model. The excitatory and the inhibitory subpopulations are recurrently coupled and receive external background inputs $\mu_{E,I}^{\text{ext}}$ and time-varying external control currents *u*_*E,I*_(*t*).

The model consists of two recurrently coupled excitatory (*E*) and inhibitory (*I*) populations (cf. Cakan and Obermayer, 2020), whose activities are measured in terms of their average firing rates *r*_*E*_(*t*) and *r*_*I*_(*t*) (see Figure 1). Both populations receive static background inputs $\mu_{E}^{\text{ext}}$ and $\mu_{I}^{\text{ext}}$ and time-varying external control inputs *u*_*E*_(*t*) and *u*_*I*_(*t*).

The model is derived from a network of excitatory and inhibitory EIF neurons under the assumption of sparse and random connectivity to neurons of the same or opposite type and in the limit of an infinite number of neurons. All parameters and variables are biophysically grounded.

#### 2.1.1. The spiking neuron model

In a network of identical EIF neurons, the dynamics of the membrane voltage of the *i*th neuron is described by (cf. Augustin et al., 2017; Cakan and Obermayer, 2020).


(1) $$\begin{array}{l} C\cdot \frac{dV_{i}}{dt}=I_{i,\text{ion}}\left(V_{i}\right)+I_{i}\left(t\right)+\mu_{i}^{\text{ext}}\left(t\right). \end{array}$$

The ion current *I*_*i*,ion_ of an EIF neuron is given by


(2) $$\begin{array}{l} I_{i,\text{ion}}\left(V_{i}\right)=g_{L}\cdot \left(E_{L}-V_{i}\left(t\right)\right)+\Delta_{T}\cdot \exp\frac{V_{i}\left(t\right)-V_{T}}{\Delta_{T}}, \end{array}$$

where *E*_*L*_, Δ_*T*_, and *V*_*T*_ are the leak reversal potential, the threshold slope factor, and the threshold voltage, respectively. Whenever the membrane voltage reaches or exceeds the spike threshold *V*_*s*_, i.e., *V*_*i*_ ≥ *V*_*s*_, an action potential is generated, the membrane voltage is changed to the reset voltage *V*_*r*_, i.e., *V*_*i*_ = *V*_*r*_, and clamped for the refractory time *T*_ref_. Table 1 summarizes the numerical values of these parameters (cf. Cakan and Obermayer, 2020).

**Table 1:** Parameters of the mean-field EI EIF model (upper block) and the spiking neuron model (lower block).

**Parameter**	**Description**	**Numerical value**
*J*_*EE*_	Maximum synaptic current from *E* to *E*	2.4 mV ms-1
*J*_*EI*_	Maximum synaptic current from *I* to *E*	-3.3 mV ms-1
*J*_*IE*_	Maximum synaptic current from *E* to *I*	2.6 mV ms-1
*J*_*II*_	Maximum synaptic current from *I* to *I*	-1.6 mV ms-1
*c*_*EE*_, *c*_*IE*_	Maximum AMPA postsynaptic current (PSC) amplitude	0.3 mV ms-1
*c*_*EI*_, *c*_*II*_	Maximum GABA PSC amplitude	0.5 mV ms-1
τ_*s,E*_	Excitatory synaptic time constant	2 ms
τ_*s,I*_	Inhibitory synaptic time constant	5 ms
*C*	Membrane capacitance	200 pF
*g*_*L*_	Leak conductance	10 nS
τ_*m*_ = *C*/*g*_*L*_	Membrane time constant	20 ms
*d*_*E*_	Synaptic delay to excitatory neurons	4 ms
*d*_*I*_	Synaptic delay to inhibitory neurons	2 ms
*K*_*E*_	Mean number of excitatory inputs per neuron	800
*K*_*I*_	Mean number of inhibitory inputs per neuron	200
$\sigma_{E,I}^{\text{ext}}$	standard deviation of external input	1.5 $mV/\sqrt{ms}$
*E*_*L*_	Leak reversal potential	-65 mV
Δ_*T*_	Threshold slope factor	1.5 mV
*V*_*T*_	Threshold voltage	-50 mV
*V*_*i*_	Spike threshold	-40 mV
*V*_*r*_	Reset potential	-70 mV
*T*_ref_	Refractory time	1.5 ms

Values are taken from Cakan and Obermayer (2020).

*I*_*i*_(*t*) is the sum of synaptic currents to the *i*th neuron induced by the neural activity of the connected neurons in the network. Excitatory (*E*) and inhibitory (*I*) neurons stimulate subsequently connected neurons differently, hence the synaptic current that neuron *i* of population α, α ∈ {*E, I*} receives is given by


(3) $$\begin{array}{l} I_{i,\alpha }\left(t\right)=C\cdot \left(J_{\alpha E}s_{i,\alpha E}\left(t\right)+J_{\alpha I}s_{i,\alpha I}\left(t\right)\right). \end{array}$$

*C* denotes the membrane capacitance, and *J*_αβ_ quantifies the coupling strength, i.e., the maximum synaptic current from population β to population α when all synapses are active. The fraction *s*_*i*,αβ_ of active synapses is determined by


(4) $$\begin{array}{l} \frac{ds_{i,\alpha \beta }}{dt}=-\frac{s_{i,\alpha \beta }}{\tau_{s,\beta }}+\frac{c_{\alpha \beta }}{J_{\alpha \beta }}\left(1-s_{i,\alpha \beta }\right)\underset{j}{\sum }G_{ij}\underset{k}{\sum }\delta \left(t-t_{j}^{k}-d_{\alpha }\right), \end{array}$$

where τ_*s*,β_ is the synaptic time constant. We sum over all spikes *k* that neuron *j* of population β emits and that are received by neuron *i* of population α after the time delay *d*_α_. *G* is a random binary connectivity matrix, i.e., *G*_*ij*_ = 1 if neuron *j* is coupled to neuron *i* and *G*_*ij*_ = 0 else.

Each neuron in the network receives a noisy background current $\mu_{i}^{\text{ext}}\left(t\right)={\bar{\mu }}^{\text{ext}}+\sigma^{\text{ext}}\xi_{i}\left(t\right)$ with mean value ${\bar{\mu }}^{\text{ext}}$ and standard deviation σ^ext^, which are equal for all neurons within a population. ξ_*i*_(*t*) is a Gaussian noise process with mean zero and variance one.

#### 2.1.2. The mean-field model

In the limit of an infinitely large population, the spiking neuron model can be turned into a neural mass model by averaging the neural dynamics of all neurons of each type. One can express the fraction of active synapses connecting population β to population α in terms of its mean value ${\bar{s}}_{\alpha \beta }\left(t\right)$ and its variance $\sigma_{s,\alpha \beta }^{2}\left(t\right)$. These determine the average membrane current μ_α_(*t*) and its variance $\sigma_{\alpha }^{2}\left(t\right)$, which in turn determine the mean firing rate *r*_α_(*t*). We denote the model as the mean-field model of excitatory and inhibitory EIF neurons (mean-field EI EIF model). For a thorough derivation of the model equations, we refer to Augustin et al. (2017). We set parameters as in Cakan and Obermayer (2020) and list the numerical values in Table 1. The model variables are summarized in Table 2. In the following, we denote the vector of dynamical variables by **x**(*t*).

**Table 2:** Variables of the mean-field EI EIF model.

**Variable**	**Description**	**Unit**
*r*_α_	Mean firing rate of population α	Hz
μ_α_	Mean membrane current of population α	mV ms-1
σ_α_	Variance of membrane current of population α	$mV/\sqrt{ms}$
τ_α_	Effective timescale of population α	ms
${\bar{s}}_{\alpha \beta }$	Mean synaptic activity from population β to population α	1
σ_*s*,αβ_	Variance of synaptic activity from population β to population α	1

The system of delay differential-algebraic equations (DDAEs) that defines the model dynamics reads


(5) $$\begin{array}{l} \left(\begin{array}{c} r_{E}\left(t\right)-\Phi_{r}\left(\mu_{E},\sigma_{E}\right)\\ r_{I}\left(t\right)-\Phi_{r}\left(\mu_{I},\sigma_{I}\right)\\ \_\_\_\_\_\_\_\_\_\_\_\_\_\_\_\_\_\_\_\_\_\_\_\_\_\_\_\_\_\_\_\_\_\_\_\_\_\_\_\_\_\_\_\_\_\_\_\_\_\_\_\_\_\_\_\_\_\_\_\_\_\_\_\_\_\_\\ {\dot{\mu }}_{E}-\frac{1}{\tau_{E}\left(t\right)}\left(J_{EE}{\bar{s}}_{EE}\left(t\right)+J_{EI}{\bar{s}}_{EI}\left(t\right)+\mu_{E}^{\text{ext}}-\mu_{E}\left(t\right)\right)\\ {\dot{\mu }}_{I}-\frac{1}{\tau_{I}\left(t\right)}\left(J_{IE}{\bar{s}}_{IE}\left(t\right)+J_{II}{\bar{s}}_{II}\left(t\right)+\mu_{I}^{\text{ext}}-\mu_{I}\left(t\right)\right)\\ \sigma_{E}\left(t\right)-{\left(\frac{2J_{EE}^{2}\sigma_{s,EE}^{2}\left(t\right)\tau_{s,E}\tau_{m}}{\left(1+r_{EE}\left(t\right)\right)\tau_{m}+\tau_{s,E}}+\frac{2J_{EI}^{2}\sigma_{s,EI}^{2}\left(t\right)\tau_{s,I}\tau_{m}}{\left(1+r_{EI}\left(t\right)\right)\tau_{m}+\tau_{s,I}}+{\left(\sigma_{E}^{\text{ext}}\right)}^{2}\right)}^{\frac{1}{2}}\\ \_\_\_\_\_\_\_\_\_\_\_\_\_\_\_\_\_\_\_\_\_\_\_\_\_\_\_\_\_\_\_\_\_\_\_\_\_\_\_\_\_\_\_\_\_\_\_\_\_\_\_\_\_\_\_\_\_\_\_\_\_\_\_\_\_\_\\ \sigma_{I}\left(t\right)-{\left(\frac{2J_{IE}^{2}\sigma_{s,IE}^{2}\left(t\right)\tau_{s,E}\tau_{m}}{\left(1+r_{IE}\left(t\right)\right)\tau_{m}+\tau_{s,E}}+\frac{2J_{II}^{2}\sigma_{s,II}^{2}\left(t\right)\tau_{s,I}\tau_{m}}{\left(1+r_{II}\left(t\right)\right)\tau_{m}+\tau_{s,I}}+{\left(\sigma_{I}^{\text{ext}}\right)}^{2}\right)}^{\frac{1}{2}}\\ \tau_{E}\left(t\right)-\Phi_{\tau }\left(\mu_{E},\sigma_{E}\right)\\ \_\_\_\_\_\_\_\_\_\_\_\_\_\_\_\_\_\_\_\_\_\_\_\_\_\_\_\_\_\_\_\_\_\_\_\_\_\_\_\_\_\_\_\_\_\_\_\_\_\_\_\_\_\_\_\_\_\_\_\_\_\_\_\_\_\_\\ \tau_{I}\left(t\right)-\Phi_{\tau }\left(\mu_{I},\sigma_{I}\right)\\ {\dot{\bar{s}}}_{EE}+\frac{{\bar{s}}_{EE}\left(t\right)}{\tau_{s,E}}-\left(1-{\bar{s}}_{EE}\left(t\right)\right)\cdot \frac{r_{EE}\left(t\right)}{\tau_{s,E}}\\ {\dot{\bar{s}}}_{EI}+\frac{{\bar{s}}_{EI}\left(t\right)}{\tau_{s,I}}-\left(1-{\bar{s}}_{EI}\left(t\right)\right)\cdot \frac{r_{EI}\left(t\right)}{\tau_{s,I}}\\ {\dot{\bar{s}}}_{IE}+\frac{{\bar{s}}_{IE}\left(t\right)}{\tau_{s,E}}-\left(1-{\bar{s}}_{IE}\left(t\right)\right)\cdot \frac{r_{IE}\left(t\right)}{\tau_{s,E}}\\ {\dot{\bar{s}}}_{II}+\frac{{\bar{s}}_{II}\left(t\right)}{\tau_{s,I}}-\left(1-{\bar{s}}_{II}\left(t\right)\right)\cdot \frac{r_{II}\left(t\right)}{\tau_{s,I}}\\ {\dot{\sigma }}_{s,EE}^{2}-\frac{1}{\tau_{s,E}^{2}}\left({\left(1-{\bar{s}}_{EE}\left(t\right)\right)}^{2}\cdot \rho_{EE}\left(t\right)+\left(\rho_{EE}\left(t\right)-2\tau_{s,E}\left(r_{EE}\left(t\right)+1\right)\right)\cdot \sigma_{s,EE}^{2}\left(t\right)\right)\\ {\dot{\sigma }}_{s,EI}^{2}-\frac{1}{\tau_{s,I}^{2}}\left({\left(1-{\bar{s}}_{EI}\left(t\right)\right)}^{2}\cdot \rho_{EI}\left(t\right)+\left(\rho_{EI}\left(t\right)-2\tau_{s,I}\left(r_{EI}\left(t\right)+1\right)\right)\cdot \sigma_{s,EI}^{2}\left(t\right)\right)\\ {\dot{\sigma }}_{s,IE}^{2}-\frac{1}{\tau_{s,E}^{2}}\left({\left(1-{\bar{s}}_{IE}\left(t\right)\right)}^{2}\cdot \rho_{IE}\left(t\right)+\left(\rho_{IE}\left(t\right)-2\tau_{s,E}\left(r_{IE}\left(t\right)+1\right)\right)\cdot \sigma_{s,IE}^{2}\left(t\right)\right)\\ {\dot{\sigma }}_{s,II}^{2}-\frac{1}{\tau_{s,I}^{2}}\left({\left(1-{\bar{s}}_{II}\left(t\right)\right)}^{2}\cdot \rho_{II}\left(t\right)+\left(\rho_{II}\left(t\right)-2\tau_{s,I}\left(r_{II}\left(t\right)+1\right)\right)\cdot \sigma_{s,II}^{2}\left(t\right)\right) \end{array}\right)=0. \end{array}$$

The system (Equation 5) of equations consists of four blocks. The population averages *r*_α_(*t*), α ∈ {*E, I*}, of the excitatory and inhibitory rates (first block), are determined by the precomputed transfer function Φ_*r*_(μ_α_, σ_α_), which depends on the corresponding mean membrane current μ_α_ and its standard deviation σ_α_. Their dynamics are described in the second block. The membrane current μ_α_ exponentially decays with the time constant τ_α_ while the weighted sum $\underset{\beta =E,I}{\sum }J_{\alpha \beta }{\bar{s}}_{\alpha \beta }$ of mean synaptic inputs and the background current $\mu_{\alpha }^{\text{ext}}$ counteract the decay. To relate μ_α_ (given in units of mV ms-1) to a physical electric current (given in units of A), it is multiplied with the membrane capacitance *C*. The variances of the membrane currents combine the variances $\sigma_{s,\alpha \beta }^{2}$, α, β ∈ {*E, I*} of the synaptic inputs and the fixed parameter $\sigma_{\alpha }^{\text{ext}}$. *r*_αβ_ denotes the population activity received by population β from population α after the time delay *d*_β_.


(6) $$\begin{array}{l} r_{\alpha \beta }\left(t\right)=\frac{c_{\alpha \beta }}{\left|J_{\alpha \beta }\right|}K_{\beta }\tau_{s,\beta }\cdot r_{\beta }\left(t-d_{\beta }\right). \end{array}$$

The fraction $\frac{c_{\alpha \beta }}{\left|J_{\alpha \beta }\right|}$ of the maximum postsynaptic and the maximum synaptic current downscales the effect of the incoming rate *r*_β_. Each neuron of population *E* and *I* is connected to *K*_β_ neurons of population β. The third block contains the effective time constants τ_α_, which the mean membrane current of the excitatory and inhibitory population decay with. They are determined by a precomputed function Φ_τ_ that depends on μ_α_ and σ_α_. The last block defines the synaptic activities ${\bar{s}}_{\alpha \beta }$ of the recurrently coupled populations and their variances $\sigma_{s,\alpha \beta }^{2}$. ${\bar{s}}_{\alpha \beta }$ decays exponentially with the time constants τ_*s*,β_ and increases depending on the activity *r*_αβ_ transmitted from population β. The variance $\sigma_{s,\alpha \beta }^{2}$ combines the uncertainties of the different contributions to ${\bar{s}}_{\alpha \beta }$, where


(7) $$\begin{array}{l} \rho_{\alpha \beta }\left(t\right)=\frac{c_{\alpha \beta }^{2}}{J_{\alpha \beta }^{2}}K_{\beta }\tau_{s,\beta }^{2}\cdot r_{\beta }\left(t-d_{\beta }\right). \end{array}$$

Time delays enter the system through *r*_αβ_ and ρ_αβ_. We denote the system of DDAEs (see Equation 5) by


(8) $$\begin{array}{l} \text{h}\left(\dot{\text{x}}\left(t\right),\text{x}\left(t\right),\text{x}\left(t-d_{E}\right),\text{x}\left(t-d_{I}\right)\right)=0. \end{array}$$

#### 2.1.3. State space of the mean-field EI EIF model

**Figure 2:**
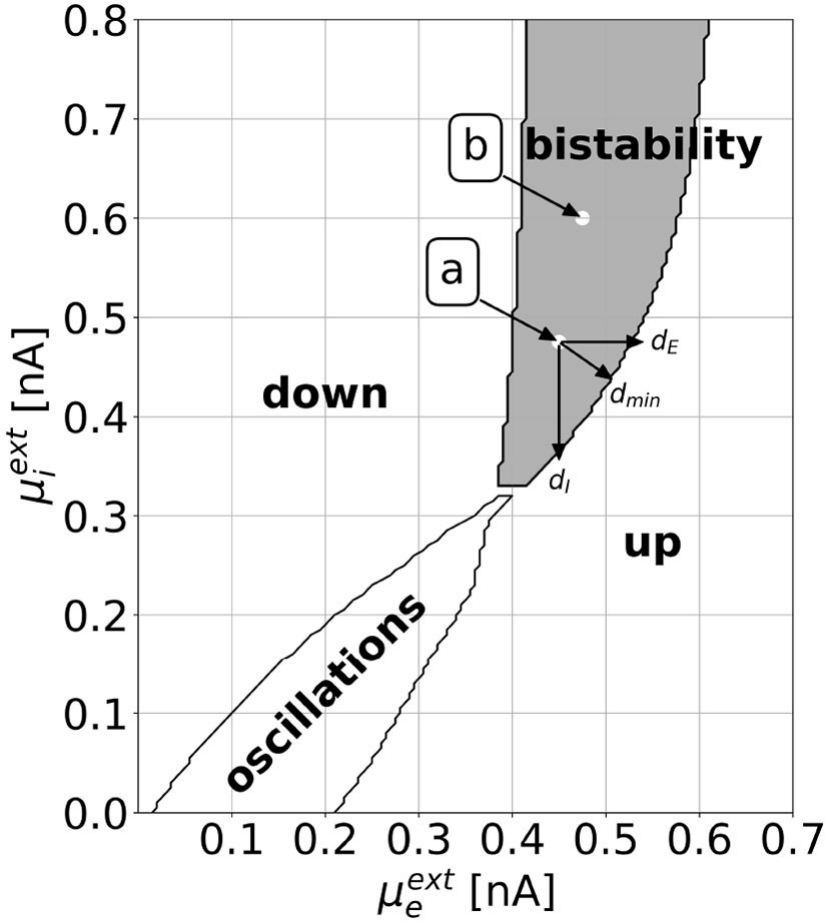
The dynamical landscape of the mean-field EI EIF model. Depending on the mean background inputs $\mu_{E}^{\text{ext}}$ and $\mu_{I}^{\text{ext}}$, we observe a down state, an up state, an oscillatory regime, or a bistable regime, where down and up states coexist. We choose two locations, which we call point a ($\mu_{E}^{\text{ext}}=0.45nA,\mu_{I}^{\text{ext}}=0.475nA$) and point b ($\mu_{E}^{\text{ext}}=0.475nA,\mu_{I}^{\text{ext}}=0.6nA$), for which we show explicit results in Section 3. We define the horizontal, vertical, and shortest distance to the regime boundary as *d*_*E*_, *d*_*I*_, and *d*_min_, respectively. This definition can be applied both for the distances to the up regime, as shown in the figure, and to the down regime.

Figure 2 shows a slice through the state space of the EI EIF model. With the parameters as defined in Table 1, one can observe all dynamically interesting phenomena by varying the external background inputs $\mu_{E}^{\text{ext}}$ and $\mu_{I}^{\text{ext}}$, which take the role of bifurcation parameters. With numerical simulations, we find a down state of constant low activity, an up state of constant high activity, a limit cycle with rate oscillations, and a bistable regime, where stable states of constant low and high activities coexist. We validate the stability of these points by numerically evaluating the Jacobian Matrix (see Supplementary section 1). Minimal and maximal values of the rates vary throughout the regimes. For a thorough analysis of the dynamics, refer to Cakan and Obermayer (2020). In this work, we focus on the bistable regime and investigate how to switch from one stable state to another. Bistability is considered an important feature for realistic models of brain dynamics as similar patterns appear in biological neural networks (Latham et al., 2000; Holcman and Tsodyks, 2006).

### 2.2. Nonlinear optimal control

#### 2.2.1. The control setting

Optimal control theory enables us to find a control function **u**(*t*) that affects a dynamical system in an efficient way to reach a target state $\tilde{\text{x}}\left(t\right)$. We quantify the performance of the control **u**(*t*) with a cost functional. Minimal costs reflect optimality. Minimizing the cost functional is a constrained optimization problem. In a controlled setting, the system of DDAEs (see Equations 5 and 8) depends on the external control function **u**(*t*),


(9) $$\begin{array}{l} \text{h}\left(\dot{\text{x}}\left(t\right),\text{x}\left(t\right),\text{x}\left(t-d_{E}\right),\text{x}\left(t-d_{I}\right),\text{u}\left(t\right)\right)=0. \end{array}$$

We denote the total cost functional by $F\left(\text{x}\left(t,\text{u}\left(t\right)\right),\tilde{\text{x}}\left(t\right),\text{u}\left(t\right)\right)$. It depends on the state vector **x**(*t*, **u**(*t*)), the target state $\tilde{\text{x}}\left(t\right)$, and the control **u**(*t*). The total cost F is the weighted sum of three contributions (Casas et al., 2015),


(10) $$\begin{array}{l} F\left(\text{x}\left(t,\text{u}\left(t\right)\right),\tilde{\text{x}}\left(t\right),\text{u}\left(t\right)\right)=F_{P}\left(\text{x}\left(t,\text{u}\left(t\right)\right),\tilde{\text{x}}\left(t\right)\right)+W_{1}\cdot F_{1}\left(\text{u}\left(t\right)\right)\\ +W_{2}\cdot F_{2}\left(\text{u}\left(t\right)\right). \end{array}$$

The precision cost *F*_*P*_ measures how accurately the target state $\tilde{\text{x}}\left(t\right)$ is reached. It is defined as the integral over the squared difference of the actual state **x**(*t*) and the target state $\tilde{\text{x}}\left(t\right)$,


(11) $$\begin{array}{l} F_{P}=\frac{1}{2}\overset{t_{1}}{\underset{t_{0}}{\int }}{\left\|\text{x}\left(t,\text{u}\left(t\right)\right)-\tilde{\text{x}}\left(t\right)\right\|}^{2}dt. \end{array}$$

Imprecision is penalized in a time interval [*t*_0_, *t*_1_]. In this study, [*t*_0_, *t*_1_] is at the end of the control interval [0, *T*]. We denote the integrand by $f_{P}=\frac{1}{2}{\left\|\text{x}-\tilde{\text{x}}\right\|}^{2}$. The “efficiency” of the control input is quantified by one cost functional that uses the *L*^1^-norm, *F*_1_, and one cost functional that uses the *L*^2^-norm, *F*_2_. In the literature, former is often referred to as the “sparsity cost” and the latter as the “energy cost.” The *F*_1_-cost is defined as Casas et al. (2015).


(12) $$\begin{array}{l} F_{1}=\overset{\dim\text{u}}{\underset{i=1}{\sum }}\sqrt{\overset{T}{\underset{0}{\int }}u_{i}^{2}dt}. \end{array}$$

By integrating over the squared components of the control signal and taking the square root for each dimension individually before summing over the input dimensions, this cost functional enforces a small number of control input channels with non-zero control strength. The *F*_2_-cost measures the total strength of the control signal and enforces small absolute values. It is given by


(13) $$\begin{array}{l} F_{2}=\frac{1}{2}\overset{T}{\underset{0}{\int }}{\|\text{u}\left(t\right)\|}^{2}dt. \end{array}$$

The optimal control u^*^(*t*) is defined as the control with minimal cost,


(14) $$\begin{array}{l} {\text{u}}^{*}\left(t\right)={\arg\min}_{\text{u}}F\left(\text{x}\left(t,\text{u}\left(t\right)\right),\tilde{\text{x}}\left(t\right),\text{u}\left(t\right)\right). \end{array}$$

By choosing the weights *W*_1_ and *W*_2_ appropriately, one can enforce different characteristics of the optimal control solution.

#### 2.2.2. The optimal control algorithm

We compute the optimal control with a gradient descent algorithm. The gradient of the cost functional with respect to the control is obtained from the adjoint method (we provide an explicit derivation in the Supplementary section 2, based on Göllmann et al., 2009; Biegler, 2010). It is given by


(15) $$\begin{array}{l} \nabla_{\text{u}}F=\overset{T}{\underset{0}{\int }}\nabla_{\text{u}}f+\lambda^{T}\cdot D_{\text{u}}\text{h}dt. \end{array}$$

**h** denotes the system dynamics (see Equations 5 and 8), *D*_**u**_ is the Jacobian matrix with respect to the control, **λ**(*t*) is the so-called adjoint state, and the components of ∇_**u**_*f* = *W*_1_·∇_**u**_*f*_1_ + *W*_2_·∇_**u**_*f*_2_ are given by Casas et al. (2015).


(16) $$\begin{array}{l} {\left(\nabla_{u}f_{1}\right)}_{\alpha }=\{\begin{array}{ll} \frac{u_{\alpha }}{\sqrt{\int_{0}^{T}|u_{\alpha }{|}^{2}dt}}dt & \text{if }\int_{0}^{T}|u_{\alpha }{|}^{2}\neq 0,\\ 0 & \text{else} \end{array},\;\alpha \in \{E,I\},\\ {\left(\nabla_{u}f_{2}\right)}_{\alpha }=|u_{\alpha }|,\;\alpha \in \{E,I\}. \end{array}$$

The adjoint state **λ**(*t*) is defined by the differential equation


(17) $$\begin{array}{l} \nabla_{\text{x}}f_{P}+\lambda^{T}\left(D_{\text{x}}\text{h}+\chi_{\left[0,T-d_{E}\right]}D_{{\text{x}}_{E}}\text{h}+\chi_{\left[0,T-d_{I}\right]}D_{{\text{x}}_{I}}\text{h}\right)-{\dot{\lambda }}^{T}D_{\dot{\text{x}}}\text{h}=0. \end{array}$$

with the final condition **λ**(*T*) = 0. In Equation (17), χ_[_*t*__*a*_,*t*_*b*_]_ denotes the indicator function on the interval [*t*_*a*_, *t*_*b*_]. *D*_**x**_, *D*_**x**_*E*__, *D*_**x**_*I*__, and $D_{\dot{\text{x}}}$ are the Jacobian matrices with respect to the state variable at time *t* (i.e., **x**(*t*)), at time *t* − *d*_*E*_ (i.e., **x**(*t* − *d*_*E*_)), at time *t* − *d*_*I*_ (i.e., **x**(*t* − *d*_*I*_)), and the Jacobian matrix with respect to the derivative of the state variable (i.e., $\dot{\text{x}}\left(t\right)$).

**Figure 3:**
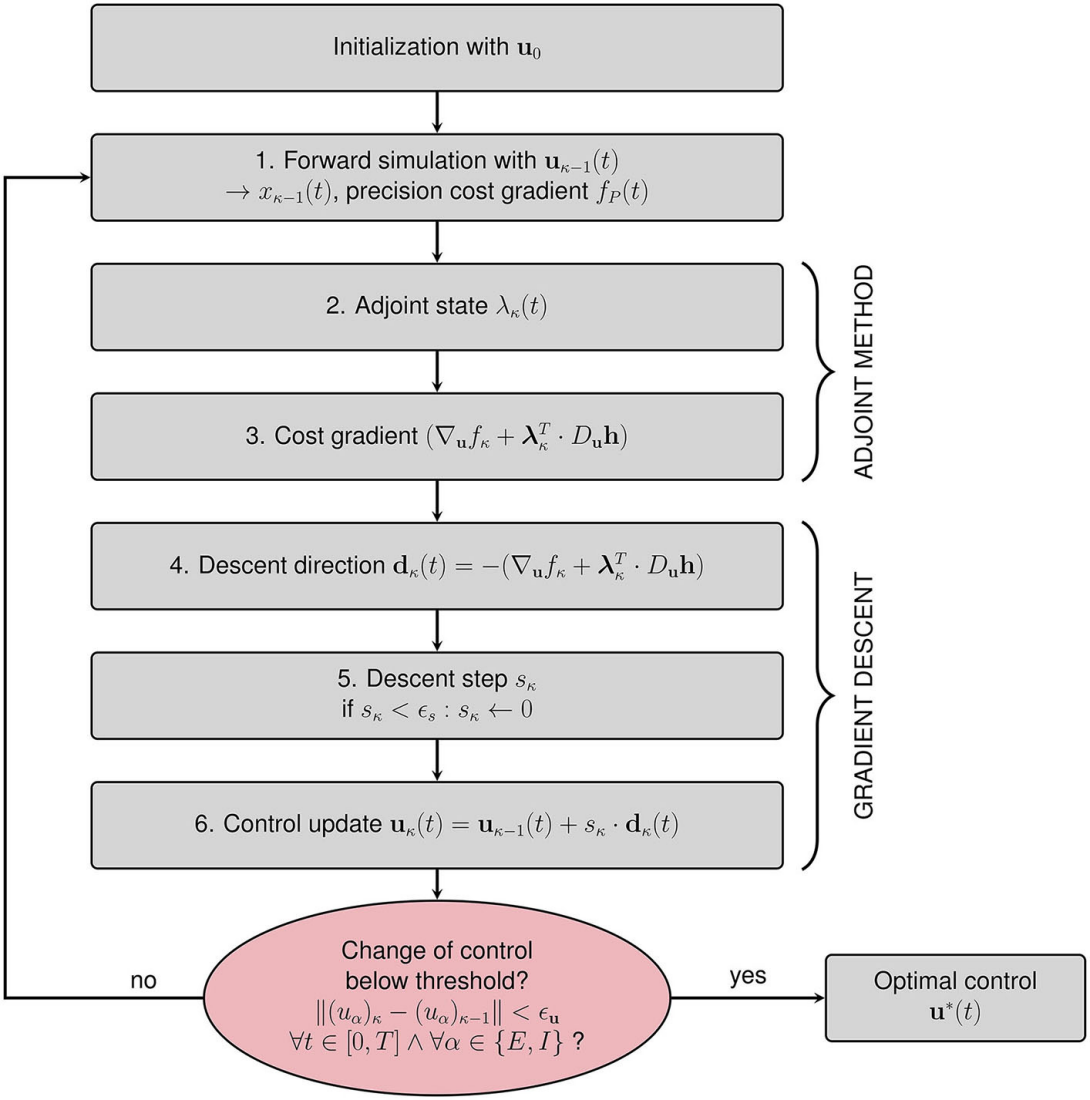
Flowchart summarizing the gradient descent procedure for computing the optimal control u^*^(*t*). After initializing the algorithm with an initial guess for the control **u**_0_(*t*), six steps are performed within each iteration. The algorithm terminates if the change of control between subsequent iterations is below a predefined threshold value ϵ_**u**_ in all components and for all points of time.

The iterative algorithm for the calculation of the optimal control u^*^(*t*) is given in Figure 3. After initialization with a first guess **u**_0_ for the optimal control (see Section 2.2.4.1), the steps in the κth iteration are as follows:

1. Perform a forward simulation using **u**_κ−1_(*t*) to obtain all dynamical variables **x**_κ−1_(*t*).
2. Compute the adjoint state **λ**_κ_(*t*) by solving Equation (17) backward in time with the initial condition **λ**_κ_(*T*) = 0.
3. Compute the gradient $\left(\nabla_{\text{u}}f_{\kappa }+\lambda_{\kappa }^{T}\cdot D_{\text{u}}\text{h}\right)$.
4. Set the descent direction ${\text{d}}_{\kappa }\left(t\right)=-\left(\nabla_{\text{u}}f_{\kappa }+\lambda_{\kappa }^{T}\cdot D_{\text{u}}\text{h}\right)$.
5. Find an appropriate step size *s*_κ_ such that **u**_κ_(*t*) = **u**_κ−1_(*t*)+*s*_κ_·**d**_κ_(*t*) outperforms **u**_κ−1_(*t*) in terms of total costs. We start by multiplying **d**_κ_(*t*) with a step size *s*_κ_ = 10. We halve *s*_κ_ and evaluate the cost resulting from **u**_κ−1_(*t*)+*s*_κ_·**d**_κ_(*t*) until we find the cost minimum. We choose this step size *s*_κ_. The bisection algorithm returns *s*_κ_ = 0 if the step size falls below a threshold value ϵ_*s*_ to avoid infinite loops.
6. Update the control **u**_κ_(*t*) = **u**_κ−1_(*t*)+*s*_κ_·**d**_κ_(*t*).

We terminate the iteration if the change of the control **u**_κ_(*t*)−**u**_κ−1_(*t*) is below a threshold value ϵ_**u**_ in all components and for all points of time.

#### 2.2.3. Optimal control of the mean-field EI EIF model

We add time-varying functions *u*_*E*_(*t*) and *u*_*I*_(*t*) to the differential equations that define the membrane currents of the mean-field EI EIF model (see Equation 5 and Figure 1).


(18) $$\begin{array}{l} \begin{array}{c} {\dot{\mu }}_{E}=\frac{1}{\tau_{E}\left(t\right)}\left(\sum_{\alpha =E,I}J_{E\alpha }{\bar{s}}_{E\alpha }\left(t\right)-\mu_{E}\left(t\right)+\mu_{E}^{\text{ext}}\right)\to \\ {\dot{\mu }}_{E}=\frac{1}{\tau_{E}\left(t\right)}\left(\underset{\alpha =E,I}{\sum }J_{E\alpha }{\bar{s}}_{E\alpha }\left(t\right)-\mu_{E}\left(t\right)+\mu_{E}^{\text{ext}}+u_{E}\left(t\right)\right)\\ {\dot{\mu }}_{I}=\frac{1}{\tau_{I}\left(t\right)}\left(\underset{\alpha =E,I}{\sum }J_{I\alpha }{\bar{s}}_{I\alpha }\left(t\right)-\mu_{I}\left(t\right)+\mu_{I}^{\text{ext}}\right)\to \\ {\dot{\mu }}_{I}=\frac{1}{\tau_{I}\left(t\right)}\left(\underset{\alpha =E,I}{\sum }J_{I\alpha }{\bar{s}}_{I\alpha }\left(t\right)-\mu_{I}\left(t\right)+\mu_{I}^{\text{ext}}+u_{I}\left(t\right)\right). \end{array} \end{array}$$

Note that the control inputs are measured in units of mV ms-1. However, they can be converted to currents measured in units of A by multiplication with the membrane capacitance *C*. We will present our results in units of nA.

We compute and investigate the optimal control for the tasks of driving the EI EIF model from the down to the up state and vice versa, for either *L*^1^- or *L*^2^-constraints, and for various parameter combinations $\left(\mu_{E}^{\text{ext}},\mu_{I}^{\text{ext}}\right)$ in the bistable regime (see Figure 2). This yields four tasks per parameter combination:

1. Down state → up state, *L*^1^-constraints: DU1-task,
2. Down state → up state, *L*^2^-constraints: DU2-task,
3. Up state → down state, *L*^1^-constraints: UD1-task,
4. Up state → down state, *L*^2^-constraints: UD2-task.

The observable physical quantity of the mean-field EI EIF model is the rate *r*_α_, α ∈ {*E, I*}, which, in the stable target state, does not depend on time. We observe that a state transition of *r*_*E*_ is always accompanied by a transition of *r*_*I*_. Therefore, we define the target state $\tilde{\text{x}}\left(t\right)=\tilde{r}$ *via* the mean rate of the excitatory population only, which unambiguously characterizes this target state. We chose a time window [0, *T*], during which control is active and penalize the deviation of the excitatory rate from its target value during an interval [*t*_0_, *T*], *t*_0_ ≥ 0. When *t*_0_ is small, we can investigate optimal transitions with time constraints.

We apply either *L*^1^-constraints ($W_{1}=1\cdot \frac{1}{As^{5/2}},W_{2}=0\cdot \frac{1}{A^{2}s^{3}}$) to investigate, to which population the application of control is more efficient, or *L*^2^-constraints ($W_{1}=0\cdot \frac{1}{As^{5/2}},W_{2}=1\cdot \frac{1}{A^{2}s^{3}}$) to investigate the effect of enforcing low amplitudes. The corresponding total cost reads


(19) $$\begin{array}{l} \begin{array}{c} F_{1}\left(\text{x},\text{u}\right)=F_{P}+W_{1}\cdot F_{1}=\frac{1}{2}\frac{1}{T-t_{0}}\overset{T}{\underset{t_{0}}{\int }}{r_{E}\left(t\right)-\tilde{r}}^{2}dt\\ +W_{1}\cdot \underset{\alpha =E,I}{\sum }\sqrt{\overset{T}{\underset{0}{\int }}u_{\alpha }^{2}dt},\text{or}\\ F_{2}\left(\text{x},\text{u}\right)=F_{P}+W_{2}\cdot F_{2}=\frac{1}{2}\frac{1}{T-t_{0}}\overset{T}{\underset{t_{0}}{\int }}{r_{E}\left(t\right)-\tilde{r}}^{2}dt\\ +\frac{W_{2}}{2}\overset{T}{\underset{0}{\int }}{\text{u}}^{2}dt. \end{array} \end{array}$$

To make results better comparable across different lengths of the time window of penalization *T* − *t*_0_, we multiply the precision cost with its inverse $\frac{1}{T-t_{0}}$.

We present results that investigate optimal transitions with or without time restrictions. For the former (presented in Sections 3.1, 3.2, and 3.3), we define the control time *T* = *500ms* and the precision measurement onset time *t*_0_ = *480ms*. This is significantly longer than the duration over which the optimal control signal has a finite value and enables smooth transitions without major discontinuities or other finite-size effects (see Section 3.4). Throughout the bistable regime, we find that under optimal control the target states are reached before the precision measurement starts, such that the precision cost *F*_*P*_ is negligibly small. For transitions under time constraints (presented in Section 3.4), we decrease both the simulation duration *T* and the precision measurement onset time *t*_0_ from *T* = *500ms* and *t*_0_ = *480ms* to *T* = *20ms* and *t*_0_ = *0ms* stepwise, such that *T* − *t*_0_ = *20ms* remains constant.

#### 2.2.4. Initialization

Gradient descent methods in general are only guaranteed to converge to a local optimum. Whether this optimum also corresponds to a global optimum of the cost depends on the initialization **u**_0_ of the control.

##### 2.2.4.1. Initialization for long transition times

For investigations with *T* = *500ms*, we find optimal control signals that lead to vanishing precision costs, *F*_*P*_ ≈ 0. Therefore, the final control result does not depend on the weight *W*_*j*_, as long as *W*_*j*_ is below a threshold value that we denote by *W*_*j*,max_. Beyond *W*_*j*,max_, it is less costly to be imprecise and stay in the initial state than to intervene and change the state, and the algorithm will return the zero control signal **u**(*t*) = 0. *W*_*j*_ determines the relative weight of ∇_**u**_*f*_*j*_ (i.e., the gradient of the *L*^1^- or *L*^2^-cost; first term in Equation 15) and $\lambda^{T}\cdot D_{\text{u}}\text{h}$ (resulting from the precision measurement; the second term in Equation 15). During optimization, the speed of convergence may vary with the choice of *W*_*j*_. The algorithm convergences relatively fast if we frequently change *W*_*j*_ to a randomly chosen number between 0 and *W*_*j*,max_.

We denote the components of the control vector by **u**(*t*) = (*u*_*E*_(*t*), *u*_*I*_(*t*)). For the down-to-up switching tasks, we define three initializations:


(20) $$\begin{array}{l} 1.\\ \;{\left(u_{0}\right)}_{E}\;=\{\begin{array}{ll} 0 & \text{for }t\;<\;210\text{ ms}\\ 0.4\text{ nA} & \text{for }210\text{ ms }\leq \;t\;\leq \;270\text{ ms}\\ 0 & \text{for }t\;>\;270\text{ ms} \end{array}\\ \;{\left(u_{0}\right)}_{I}\;=\;0 \end{array}$$

(21) $$\begin{array}{l} 2.\\ \;{\left(u_{0}\right)}_{E}\;=0\\ \;{(u_{0})}_{I}\;=\{\begin{array}{ll} 0 & \text{for }t\;<\;210\text{ ms}\\ -0.4\text{ nA} & \text{for }210\text{ ms }\leq \;t\;\leq \;270\text{ ms}\\ 0 & \text{for }t\;>\;270\text{ ms} \end{array} \end{array}$$

(22) $$\begin{array}{l} 3.\\ \;{\left(u_{0}\right)}_{E}\;=\{\begin{array}{ll} 0 & \text{for }t\;<\;210\text{ ms}\\ 0.4\text{ nA} & \text{for }210\text{ ms }\leq \;t\;\leq \;270\text{ ms}\\ 0 & \text{for }t\;>\;270\text{ ms} \end{array}\\ \;{\left(u_{0}\right)}_{I}\;=\{\begin{array}{ll} 0 & \text{for }t\;<\;210\text{ ms}\\ -0.4\text{ nA} & \text{for }210\text{ ms }\leq \;t\;\leq \;270\text{ ms}\\ 0 & \text{for }t\;>\;270\text{ ms} \end{array} \end{array}$$

These are rectangle pulses centered at $\frac{t_{0}}{2}=240ms$. For the up-to-down switching tasks, we multiply with −1. For each of these initializations, the algorithm converges to a pulse-shaped control signal. Depending on the task and the state space parameters $\left(\mu_{E}^{\text{ext}},\mu_{I}^{\text{ext}}\right)$, all three initializations might lead to the same or two different results. In the latter case, these correspond to local optima. We validate that the algorithm returns identically shaped control signals if initialized differently (e.g., gaussian function in *u*_*E*_, *u*_*I*_, both, zero, etc.). However, shifting signals in time is computationally very time-consuming, in particular, if initializations are centered close to *t* = 0 or *t* = *t*_0_.

For each of these **u**_0_(*t*), we compute the optimal control as follows:

1. We perform ten iterations with $W_{1}=10\cdot \frac{1}{As^{5/2}}$ or $W_{2}=10\cdot \frac{1}{A^{2}s^{3}}$ allowing only control input *u*_*E*_ to the excitatory population (1. initialization), *u*_*I*_ to the inhibitory population (2. initialization), or control inputs to both populations (3. initialization).
2. We allow control inputs to both populations.
3. We set *W*_*j*_ to a random value between 0 and *W*_*j*,max_ and perform several tens of iterations. We repeat until convergence ($\epsilon_{s}=1\times 10^{-30},\epsilon_{\text{u}}=1\times 10^{-12}$, see Section 2.2.2 and Figure 3).
4. We set $W_{1}=1\cdot \frac{1}{As^{5/2}}$ or $W_{2}=1\cdot \frac{1}{A^{2}s^{3}}$ and measure the total cost of the control.

We compare the three initializations and take the result with the lowest total cost as the optimal control. This initialization yields results with peaks approximately at $\frac{t_{0}}{2}$.

##### 2.2.4.2. Initialization for reduced transition times

For point a (see Figure 2), we investigate the optimal control for shorter simulation times *T* < *500ms*. To this end, we successively reduce *T* and *t*_0_, keeping *T* − *t*_0_ = *20ms* fixed. When reducing *T*, we initialize with the optimal control signal for *T* = *500ms*, shifted back in time such that the peak remains at $\frac{t_{0}}{2}$. To avoid local optima, we also compute the optimal control for *T* = *20ms* and *t*_0_ = 0 and successively increase *T* and *t*_0_, keeping *T* − *t*_0_ = *20ms* fixed. For each optimization, we initialize with the optimal control signal of the next longest *T*. We compare the results from the two different approaches and choose the signal with the lowest total cost as the optimal control.

#### 2.2.5. Implementation and numerical computation

We implement the optimal control algorithm using neurolib (Cakan et al., 2021), an open source python simulation framework for whole-brain neural mass modeling. Neurolib offers various models of neural dynamics, including the mean-field EI EIF model described in Section 2.1. We use Euler integration with an integration step size of *dt* = *0.1ms*. We validate that this value is sufficiently small to avoid numerical inaccuracies, results are shown in the Supplementary section 3.

A graphical interface visualizes the optimal control signals and the resulting neural activity for the four state switching tasks for various parameter combinations ($\mu_{E}^{\text{ext}},\mu_{I}^{\text{ext}}$) within the bistable regime (see Figure 2). The interface is available at github.com/lenasal/Optimal_Control_GUI.

## 3. Results

### 3.1. Continuous sets of optimal control signals

**Figure 4:**
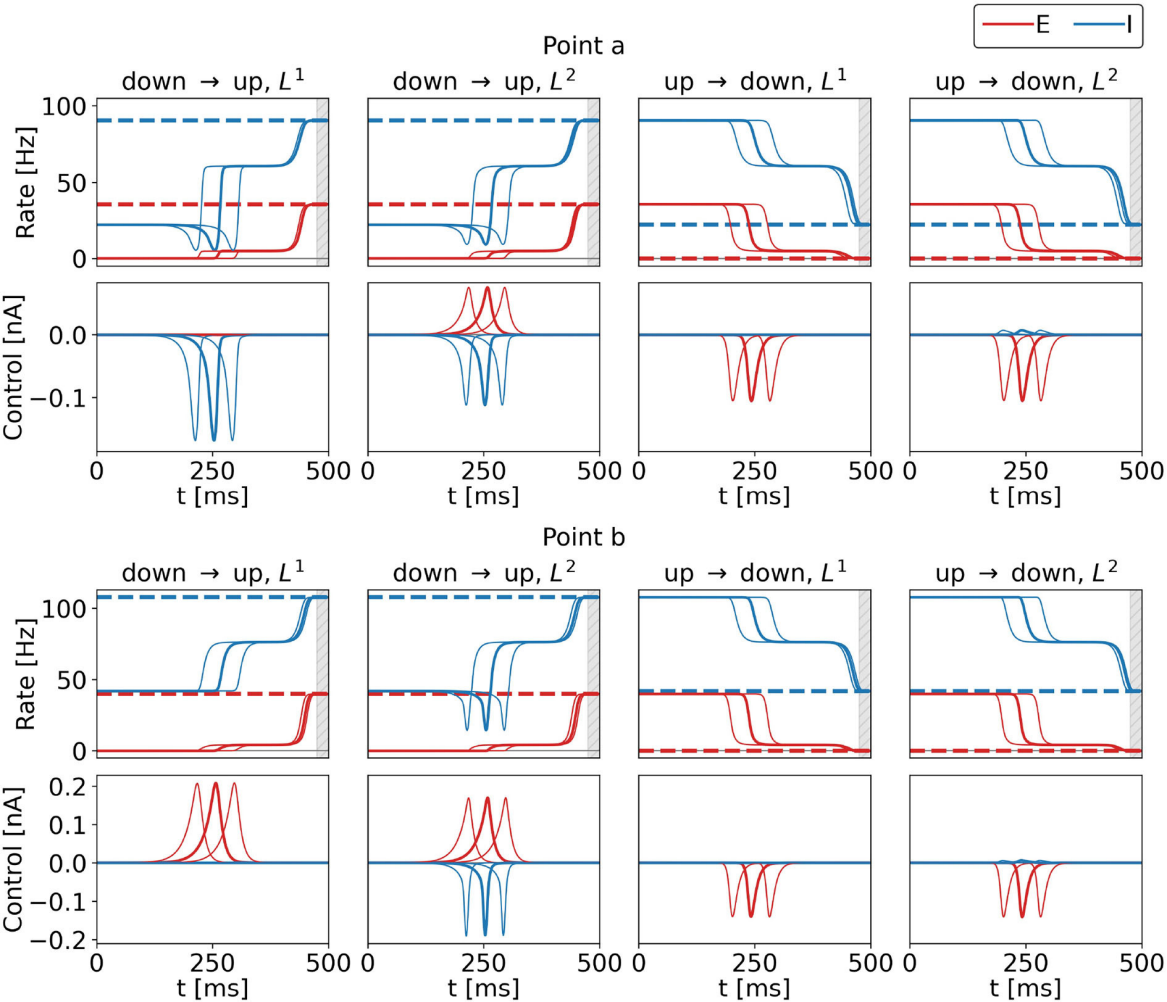
Control inputs and population rates for three different initializations for the four control tasks and for points a (top row) and b (bottom row) marked in the state space diagram of Figure 2. Bold lines show results obtained for the standard initialization, and the lines to the right (left) show results with the initialization pulse shifted by 40 ms (−40 ms). The top rows show the firing rates of the excitatory (red) and inhibitory (blue) population as a function of time, bottom rows show the corresponding optimal control currents, *u*_*E*_ in red, *u*_*I*_ in blue. From left to right, the columns show the results for the DU1-, DU2-, UD1-, and UD2-task. The respective target rates are indicated by the dashed lines. The simulation duration is *T* = 500 ms. During the last 20 ms, precision is penalized (gray shaded area). The numerical values for the costs are *F*_DU1_ = 3.3312, *F*_DU2_ = 3.5516, *F*_UD1_ = 2.1462, and *F*_UD2_ = 2.2901 at point a, and *F*_DU1_ = 5.0064, *F*_DU2_ = 10.9004, *F*_UD1_ = 2.6569, and *F*_UD2_ = 3.5209 at point b for all three initializations.

Figure 4 shows the optimal control signals and the resulting firing rates obtained from initializations as described in Section 2.2.4.1. We also show optimal control signals obtained from an initial rectangle pulse centered around 200 and 280 ms (cf. Equations 20–22). Across the three initializations, resulting costs are identical to at least five significant digits, for all four control tasks, for both points a and b. Also, there are no noteworthy differences in the control signals apart from their respective shifts by ±40 ms. We subtract the signals (shifted back by ±40 ms) from the original ones and find a difference of 31 nA at most for the two points and the four tasks. We hypothesize that there is a continuous set of optimal control signals with different peak times. For *T* → ∞, we thus expect a continuous set of global optima, where any peak time can be realized. In the following, we will present the solutions obtained from the initialization as explained in Section 2.2.4.1 only.

### 3.2. The optimal control steers the system only minimally into the target basin of attraction

When optimal control is applied, the firing rates of the excitatory and inhibitory population pass a plateau (see Figure 4, all tasks and both points). Once the control pulse is applied, the system departs from the initial state. The transition is decelerated until the system reaches the intermediate plateau state. Then, the control terminates, keeping the control effort low. As a consequence, the system relaxes and naturally accelerates toward the stable target state, which is smoothly approached. This behavior is observed for all tasks in Figure 4 and throughout the whole bistable regime (results not shown).

**Figure 5:**
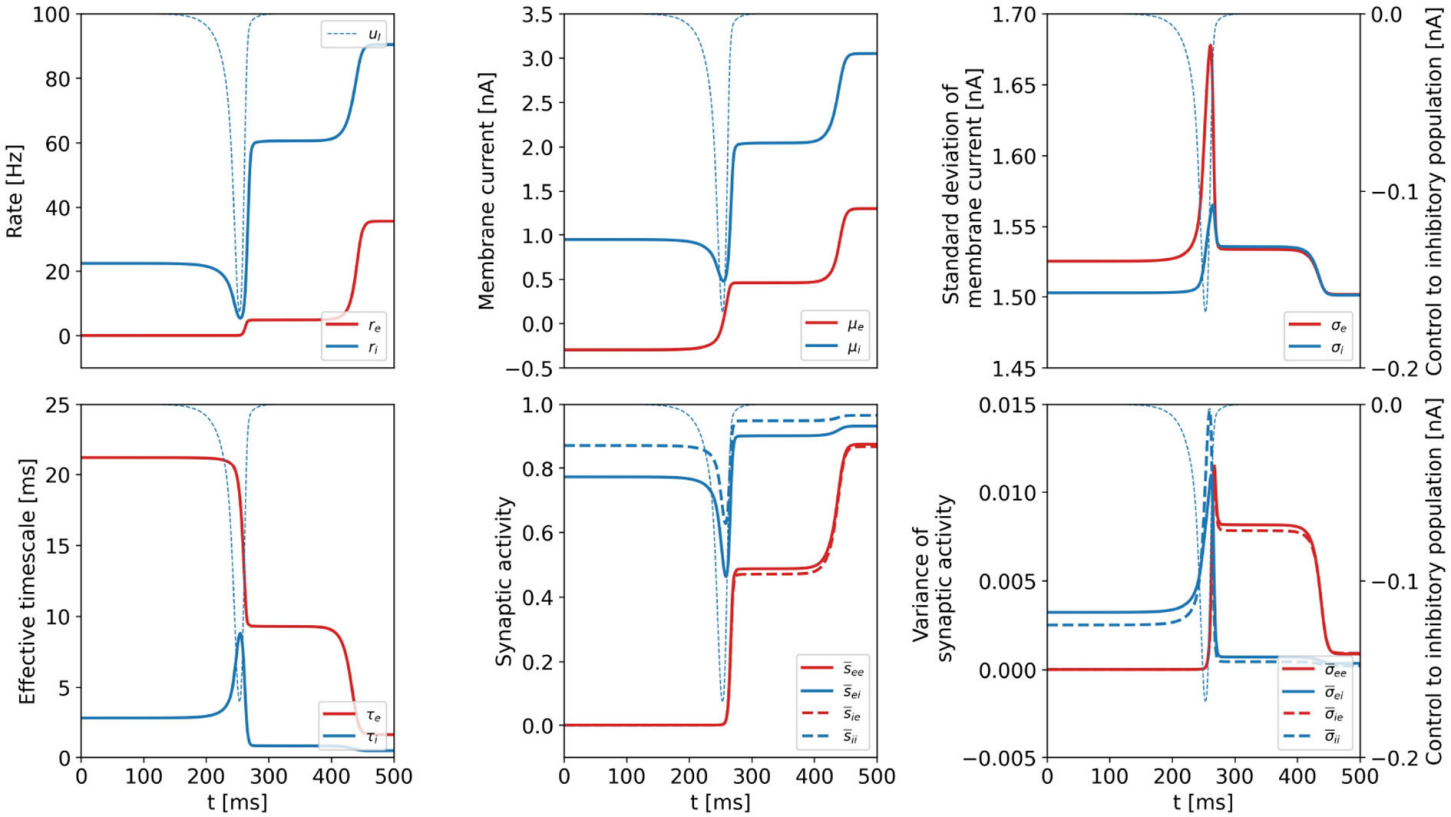
Dynamical variables as a function of time for the DU1-task, when optimal control is applied. Parameters correspond to point a shown in Figure 2. Variables related to the excitatory (inhibitory) population are plotted in red (blue). We show the optimal control input to the inhibitory population in each plot as the thin, dashed, blue line (*u*_*E*_ = 0). All dynamical variables reach a plateau state between *t*≈ 250 ms and *t* ≈ 400 ms.

We plot all dynamical variables for the DU1-task at point a in Figure 5 and verify that the constant intermediate state is a common feature of all variables. We denote the state variables at the plateau state by **x**_*P*_. As the values are constant, ${\dot{\text{x}}}_{P}\approx 0$. We hypothesize that the intermediate plateau is related to an unstable fixed point (see Supplementary section 1) that separates the basins of attraction of the initial and the final state. The control acts such that the system is steered minimally across the boundary of the basins of attraction. Once the boundary is passed, the system is certain to reach the target state without further control input.

### 3.3. Control task and state space parameters determine the optimal control

Optimal control signals are bell-shaped pulses throughout the bistable regime for all tasks. We investigate four properties of the optimal control signals:

1. **Dimensionality:** We refer to a control as one-dimensional (1d), if it is applied to one population only. For 1d control signals, either *u*_*I*_ = 0 or *u*_*E*_ = 0. If a control signal is applied to both excitatory and inhibitory populations, we call it two-dimensional (2d). 2d signals can be dominated by input to the excitatory population (max|*u*_*E*_| ≥ max|*u*_*I*_|) or by input to the inhibitory population (max|*u*_*E*_| < max|*u*_*I*_|).
2. **Amplitude:** We define the maximum of the absolute value of each control signal as its amplitude $a_{\alpha }=\underset{t}{\max}|u_{\alpha }\left(t\right)|,\alpha \in \left\{E,I\right\}$.
3. **Cost:** We investigate the effects of *L*^1^- (DU1- and UD1-task) or *L*^2^-constraints (DU2- and UD2-task). The contribution to *F*_1_ of a control signal applied to the α population is given by
  (23) $$\begin{array}{l} F_{1,\alpha }=\sqrt{\overset{T}{\underset{0}{\int }}u_{\alpha }^{2}dt}, \end{array}$$
  and the corresponding contribution to *F*_2_ by
  (24) $$\begin{array}{l} F_{2,\alpha }=\frac{1}{2}\overset{T}{\underset{0}{\int }}|u_{\alpha }\left(t\right){|}^{2}dt. \end{array}$$
4. **Width:** We define the width *w*_α_ of a control signal *u*_α_(*t*) as the duration, over which the absolute value is at least half its maximum, i.e., *w*_α_ = *t*_*w*_1__ − *t*_*w*_0__, where $\left|u_{\alpha }\left(t\right)|\geq \frac{1}{2}\cdot \max|u_{\alpha }\right|$ for *t* ∈ [*t*_*w*_0__, *t*_*w*_1__].

In the following, we denote the horizontal (vertical) distance from a selected point $\left(\mu_{E}^{\text{ext}},\mu_{I}^{\text{ext}}\right)$ to the target regime boundary by *d*_*E*_ (*d*_*I*_) and the shortest distance by *d*_min_ (see Figure 2).

**Figure 6:**
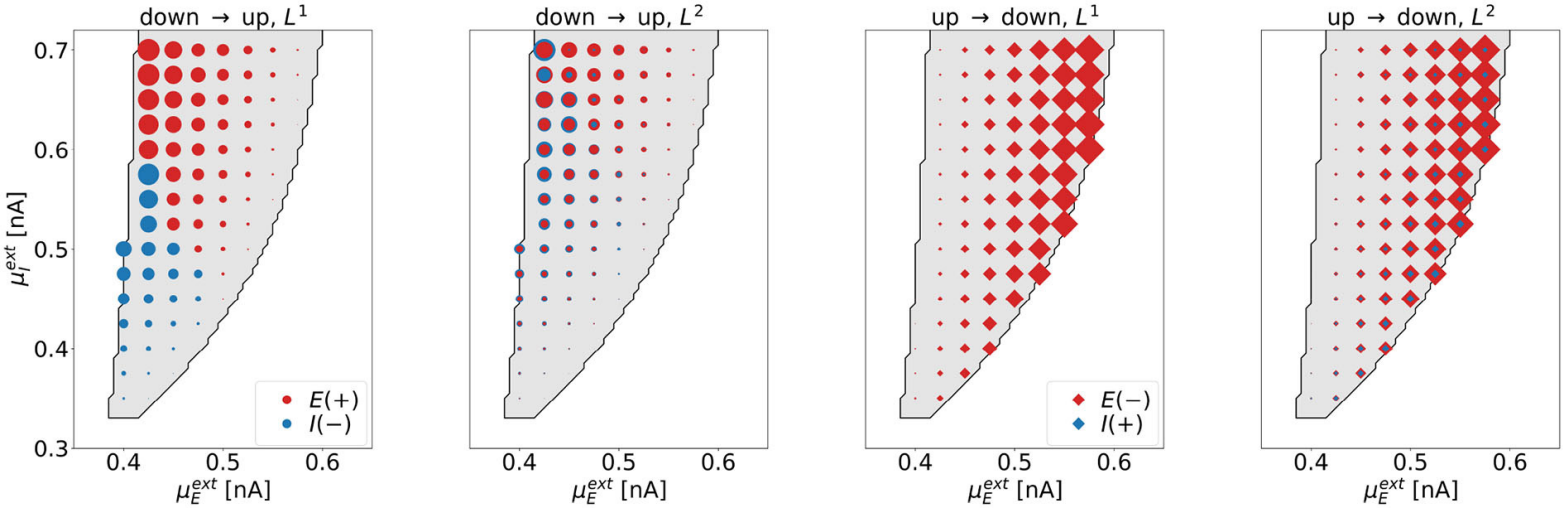
The dimensionality of the optimal control signals at selected points $\left(\mu_{E}^{\text{ext}},\mu_{I}^{\text{ext}}\right)$ in the bistable regime. The four panels correspond to the four control tasks. Each marker represents one point in state space, for which the optimal control was computed. We indicate the excitatory (inhibitory) control amplitude with red (blue) markers. The area of the markers scales with the respective amplitude of the optimal control signal. For the down-to-up tasks (first and second panel), red circles correspond to positive signals, blue circles correspond to negative signals. For the UD2-task (rightmost panel), the size of the blue diamonds was increased by a factor of 200 compared to the red diamonds to also visualize the contribution of the weak control signal *u*_*I*_.

**Dimensionality**. We investigate the dimensionality of the optimal control signals for all tasks for various parameter combinations $\left(\mu_{E}^{\text{ext}},\mu_{I}^{\text{ext}}\right)$ in the bistable regime. The results are summarized in Figure 6, where each symbol represents one point $\left(\mu_{E}^{\text{ext}},\mu_{I}^{\text{ext}}\right)$ in state space, for which the optimization was performed.

As expected, we find that *L*^1^-constraints lead to one-dimensional solutions only (DU1- and UD1-task). For the DU1-task, we find 1d control of the inhibitory population for lower and 1d control of the excitatory population for higher values of $\mu_{I}^{\text{ext}}$. For the UD1-task, all solutions show non-zero control input to the excitatory population only. Constraints resulting from applying *L*^2^-constraints lead to 2d solutions. For the DU2-task, these are dominated by input to the inhibitory population for low and by input to the excitatory population for high values of $\mu_{I}^{\text{ext}}$. For the UD2-task, all solutions are dominated by control inputs to the excitatory population.

Applying control to the excitatory (inhibitory) population is related to a shift in state-space along the $\mu_{E}^{\text{ext}}$-axis ($\mu_{I}^{\text{ext}}$-axis). The control always operates such that it moves the system toward the target regime; right or downwards for the down-to-up tasks, left or upwards for the up-to-down tasks. As a consequence, *u*_*E*_ and *u*_*I*_ always have opposite signs. Due to the almost vertical boundary toward the down regime, applying control to the inhibitory population is not efficient for the up-to-down switching tasks.

**Figure 7:**
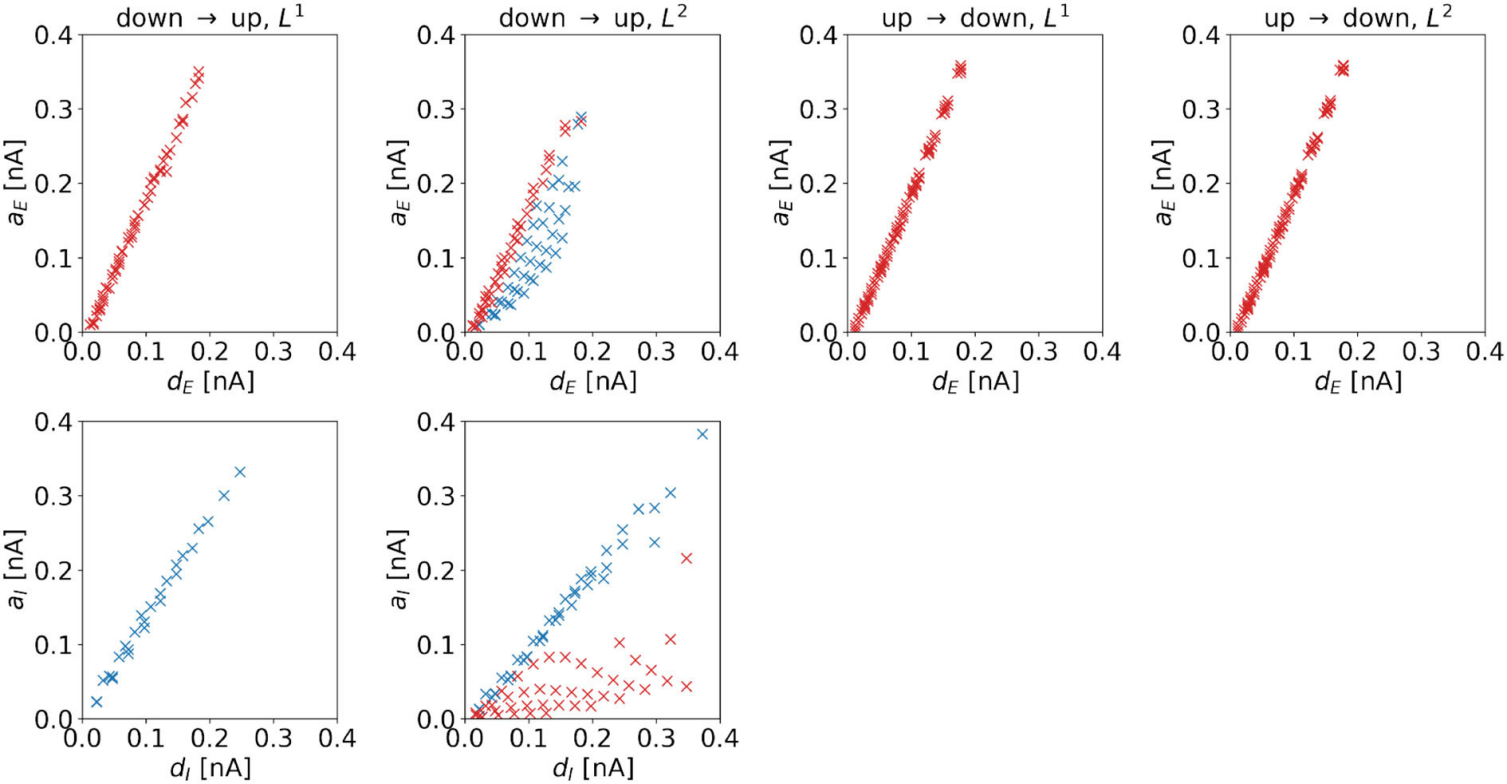
Amplitude of the optimal control signals as a function of the horizontal or vertical distance to the target regime boundary. The four columns correspond to the different tasks. We indicate 1d control of the excitatory population or 2d control with max|*u*_*E*_(*t*)| ≥ max|*u*_*I*_(*t*)| by red color and 1d control of the inhibitory population or 2d control with max|*u*_*E*_(*t*)| < max|*u*_*I*_(*t*)| by blue color. For the down-to-up switching tasks, the figures show *a*_*E*_ over *d*_*E*_ (top panel) and *a*_*I*_ over *d*_*I*_ (bottom panel). For the DU2-task, both figures include data from optimal control signals with max|*u*_*E*_(*t*)| ≥ max|*u*_*I*_(*t*)| (red markers) and max|*u*_*E*_(*t*)| < max|*u*_*I*_(*t*)| (blue markers). For the UD1- and UD2-tasks, we only show *a*_*E*_ over *d*_*E*_. Correlation coefficients of *a*_*E*_ over *d*_*E*_ are as follows: 0.9984 (DU1, *E*), 0.9935 (DU2, *E*), 0.8996 (DU2, *I*), 0.9992 (UD1), 0.9992 (UD2). Correlation coefficients of *a*_*I*_ over *d*_*I*_ are as follows: 0.9968 (DU1, *I*), 0.6279 (DU2, *E*), and 0.9909 (DU2, *I*).

**Amplitude**. The amplitude of the (dominating) control signal depends on the distance to the target regime boundary. Figure 7 shows amplitudes as a function of distances for the four control tasks. We observe linear dependencies for all cases. Comparing the top and bottom panels of the up-to-down tasks, we observe that *a*_*E*_ increases faster than *a*_*I*_ with distance, i.e., $\frac{\text{d}a_{E}}{\text{d}d_{E}}>\frac{\text{d}a_{I}}{\text{d}d_{I}}$.

For the DU2-task, we compare results with max|*u*_*E*_(*t*)| ≥ max|*u*_*I*_(*t*)| (red markers in Figure 7) to results with max|*u*_*E*_(*t*)| < max|*u*_*I*_(*t*)| (blue markers in Figure 7). For the former, *a*_*I*_ is relatively small, indicating that transitions are mainly induced by stimulation of the excitatory population. For the latter, *a*_*E*_ is relatively high, indicating that stimulation of both populations is crucial for optimal transitions.

A higher control strength, i.e., a higher amplitude, is needed to overcome a larger distance toward the target regime. Despite the highly nonlinear dynamics of the model, the required increase in amplitude scales linearly with the distance *d*_*E*_ or *d*_*I*_ in the dominating input channel.

**Figure 8:**
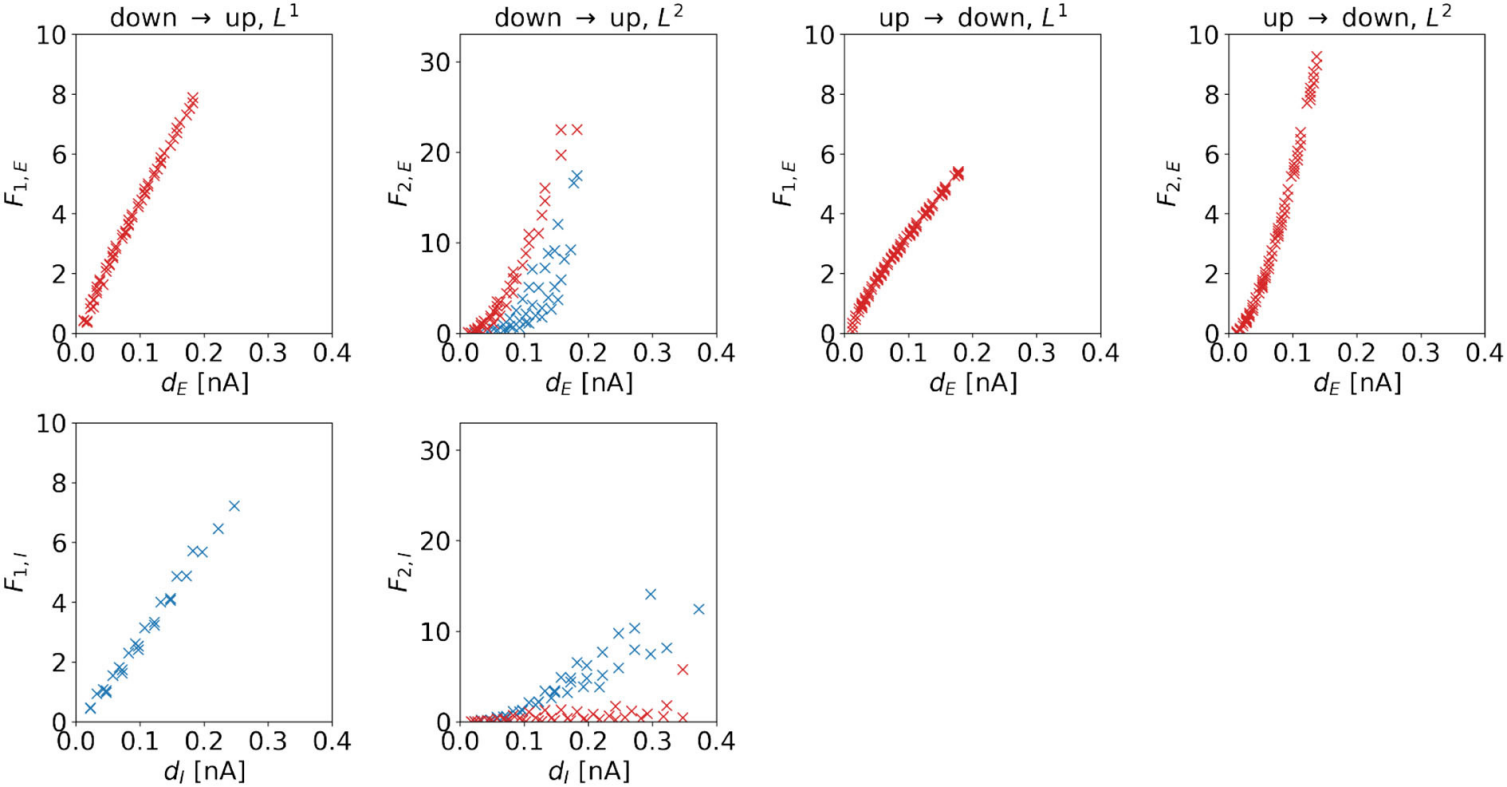
*F*_1_ and *F*_2_ of the optimal control signals as a function of the horizontal or vertical distance to the target regime boundary. The four columns correspond to the different tasks. We indicate 1d control of the excitatory population or 2d control with max|*u*_*E*_(*t*)| ≥ max|*u*_*I*_(*t*)| by red color and 1d control of the inhibitory population or 2d control with max|*u*_*E*_(*t*)| < max|*u*_*I*_(*t*)| by blue color. For the down-to-up tasks, the figure shows *F*_1,*E*_ or *F*_2,*E*_ over *d*_*E*_ (top panel) and *F*_1,*I*_ or *F*_2,*I*_ over *d*_*I*_ (bottom panel). For the DU2-task, both figures include data from optimal control signal with max|*u*_*E*_(*t*)| ≥ max|*u*_*I*_(*t*)| (red markers) and max|*u*_*E*_(*t*)| < max|*u*_*I*_(*t*)| (blue markers). For the UD1- and UD2-tasks, we only show *a*_*E*_ over *d*_*E*_. Correlation coefficients of *F*_1,*E*_ or *F*_2,*E*_ over *d*_*E*_ are as follows: 0.9980 (DU1, *E*), 0.9652 (DU2, *E*), 0.7984 (DU2, *I*), 0.9964 (UD1), and 0.9840 (UD2). Correlation coefficients of *F*_1,*I*_ or *F*_2,*I*_ over *d*_*I*_ are as follows: 0.9953 (DU1, *I*), 0.5253 (DU2, *E*), and 0.9330 (DU2, *I*).

**Cost**. The cost of the (dominating) control signal is also determined by the distance to the target regime boundary. Figure 8 shows costs as a function of distances for the four control tasks. We observe a linear dependence if *L*^1^-constraints are applied. For the DU2- and UD2-tasks, we also find a linear correlation, however, the dependence is superlinear for these control tasks. For the DU1-task, the slope of the excitatory cost is steeper than the slope for the inhibitory cost, i.e., $\frac{\text{d}F_{1,e}}{\text{d}d_{E}}>\frac{\text{d}F_{1,i}}{\text{d}d_{I}}$.

For the DU2-task, we compare results with max|*u*_*E*_(*t*)| ≥ max|*u*_*I*_(*t*)| (red markers in Figure 8) to results with max|*u*_*E*_(*t*)| < max|*u*_*I*_(*t*)| (blue markers in Figure 8). Similar to the relations found for the amplitude, we find that for the former, *F*_2,*I*_ is relatively small, whereas for the latter, *F*_2,*E*_ is relatively high.

A higher required control strength (i.e., a higher amplitude) is reflected in the corresponding cost. Due to the mathematical definition of *F*_1_ (see Equation 19, first line) and due to the fact that the amplitude scales linearly with the distance *d*_*E*_ or *d*_*I*_, the dependence of *F*_1,*e*_ or *F*_1,*i*_ on *d*_*E*_ or *d*_*I*_ is also linear. However, the definition of *F*_2_ (see Equation 19, second line) implies that, if amplitude scales linearly with distance, the dependence of the cost must be superlinear.

**Figure 9:**
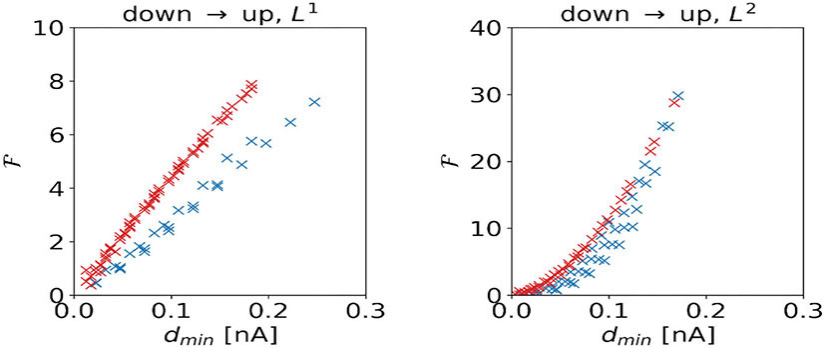
Total cost F as a function of the shortest distance *d*_min_ to the target regime boundary for the DU1- (left panel) and DU2-tasks (right panel).

We investigate the scaling of the total cost F with the shortest distance *d*_min_ to the target regime boundary. For the DU1-task, control inputs to the excitatory population produce higher total costs to overcome a certain distance to the target regime than control inputs to the inhibitory population (Figure 9, left panel). For the DU2-task, control signals dominated by inputs to the excitatory population produce higher total costs to overcome a certain distance to the target regime than control signals dominated by inputs to the inhibitory population (Figure 9, right panel).

**Figure 10:**
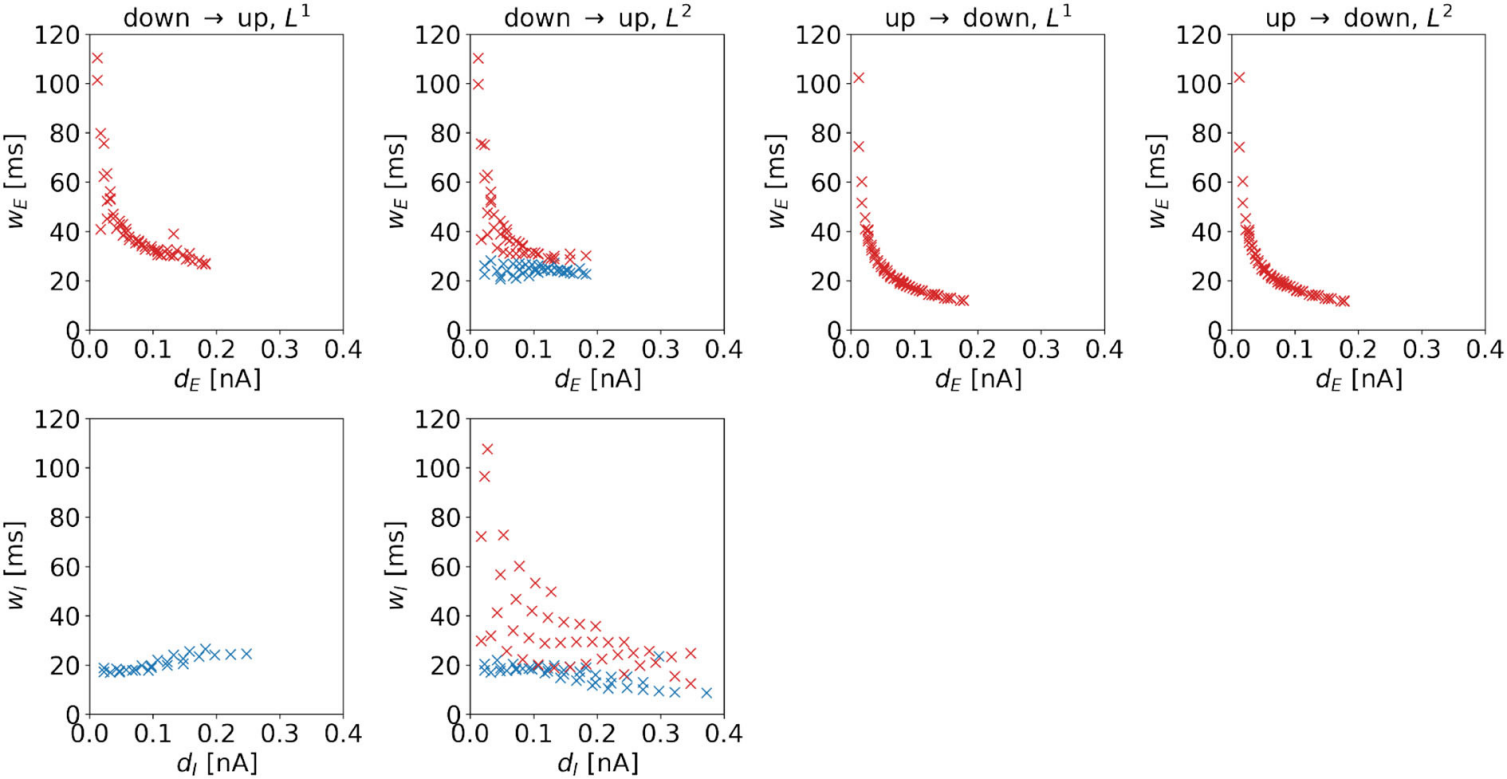
Width of the optimal control signals as a function of the horizontal or vertical distance to the target regime boundary. The four columns correspond to the different tasks. We indicate 1d control of the excitatory population or 2d control with max|*u*_*E*_(*t*)| ≥ max|*u*_*I*_(*t*)| by red color and 1d control of the inhibitory population or 2d control with max|*u*_*E*_(*t*)| < max|*u*_*I*_(*t*)| by blue color. For the down-to-up tasks, the figure shows *w*_*E*_ over *d*_*E*_ (top panel) and *w*_*I*_ over *d*_*I*_ (bottom panel). For the DU2-task, both figures include data from optimal control signal with max|*u*_*E*_(*t*)| ≥ max|*u*_*I*_(*t*)| (red markers) and max|*u*_*E*_(*t*)| < max|*u*_*I*_(*t*)| (blue markers). For the UD1- and UD2-tasks, we only show *w*_*E*_ over *w*_*E*_. Correlation coefficients of *w*_*E*_ over *d*_*E*_ are as follows: –0.7120 (DU1, *E*), –0.6479 (DU2, *E*), –0.6962 (DU2, *I*), –0.5492 (UD1), and –0.5476 (UD2). Correlation coefficients of *w*_*I*_ over *d*_*I*_ are as follows: 0.8848 (DU1, *I*), –0.6213 (DU2, *E*), and –0.6962 (DU2, *I*).

**Width**. The widths of the control signal depends on the distance to the regime boundary. For control signals dominated by inputs to the excitatory population, we observe a negative correlation (see red markers in Figure 10), i.e., such control pulses become sharper when moving away from the target regime boundary. For the DU2-task, this also holds for *w*_*I*_. In particular, *w*_*E*_ and *w*_*I*_ correlate strongly with each other, the Pearson correlation coefficient is 0.9916. For control signals dominated by inputs to the inhibitory population, we observe a positive correlation for the DU1-task (see Figure 10, first column, bottom panel), i.e., these control pulses become wider when moving away from the target regime boundary. For the DU2-task, the width of control signals dominated by inputs to the inhibitory population hardly changes with the distance to the target regime boundary.

### 3.4. Tradeoffs between transition time and cost

To investigate tradeoffs between transition time, precision cost, and strength of control, we reduce both the simulation duration *T* and the precision measurement onset time *t*_0_ from *T* = *500ms* and *t*_0_ = *480ms* to *T* = *20ms* and *t*_0_ = 0 successively, such that *T* − *t*_0_ = *20ms* remains constant (see Section 2.2.4.2).

We investigate optimal control signals for *T* ≤ *500ms* for the DU1-task at point a and for two penalization strategies. We compute the optimal control for $W_{1}=1\cdot \frac{1}{As^{5/2}}$, or for *W*_1,max_. The numerical value depends on *T* and *t*_0_.

**Figure 11:**
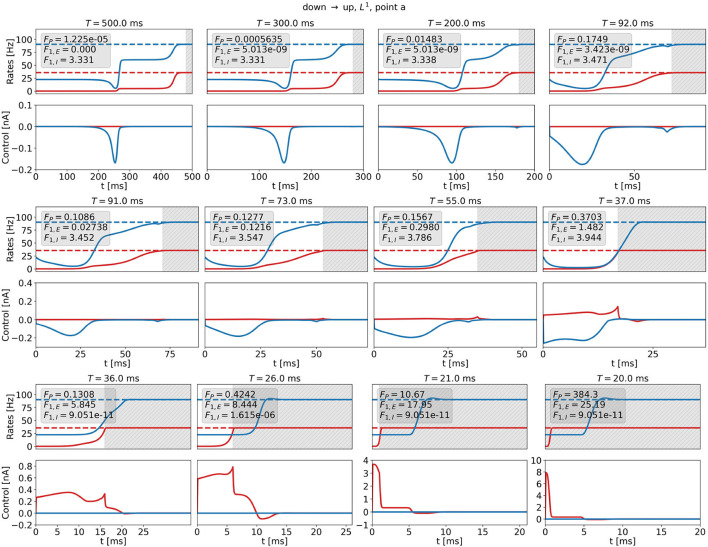
Firing rates (top panels) and optimal control signals (A) for transitions with various transition times *t*_0_ for the DU1-task at point a for $W_{1}=1\cdot \frac{1}{As^{5/2}}$. Excitatory (inhibitory) activity and control applied to the excitatory (inhibitory) population are plotted in red (blue). The gray area shows the time window of precision measurement, *T* − *t*_0_. The transition time *t*_0_ decreases from left to right and from top to bottom. The respective precision cost *F*_*P*_, and the *F*_1,*E*_- and *F*_1,*I*_-costs are given in the box of each figure.

Figure 11 shows optimal control signals and the resulting trajectories of the firing rates for several values of *T* and *t*_0_ for the DU1-task at point a for $W_{1}=1\cdot \frac{1}{As^{5/2}}$. We find three different control strategies. For large transition times, *t*_0_ ≳ *72ms, T*≳*92ms*, the optimal control remains a 1d signal to the inhibitory population (see Figure 11, top row). The cost remains almost constant with decreasing transition time, however, the plateau state becomes shorter. For intermediate transition times, *17ms* ≲ *t*_0_ ≲ *71ms, 37ms* ≲ *T* ≲ *91ms*, there is a finite contribution of *u*_*E*_ that increases when *t*_0_ becomes smaller (see Figure 11, center row). A secondary peak appears just before *t*_0_, which helps push the system toward the target state. The input to the excitatory population is much smaller than the input to the inhibitory population. For small transition times, *t*_0_ ≲ *16ms, T* ≲ *36ms*, the optimal control is a 1d signal to the excitatory population (see Figure 11, bottom row). The amplitude increases and reaches a maximum of approximately 8 nA for *t*_0_ = *0ms* (note that the scaling along both the x- and the y-axis changes). With this control strength, the firing rate of the excitatory population reaches the target state after approximately 1 ms.

**Figure 12:**
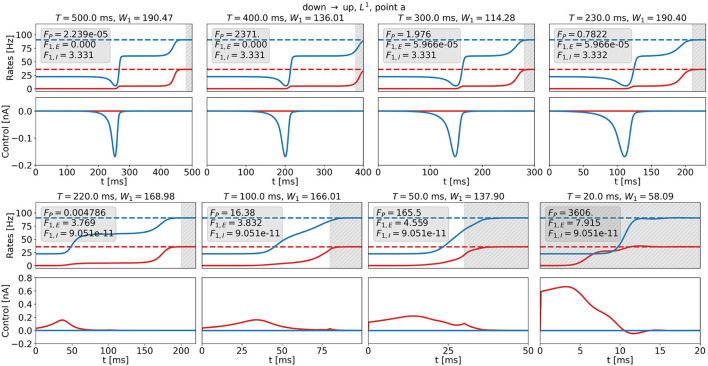
Firing rates (top panels) and optimal control signals (bottom panels) for transitions with various transition times *t*_0_ for the DU1-task at point a for *W*_1_ = *W*_1,max_. Excitatory (inhibitory) activity and control applied to the excitatory (inhibitory) population are plotted in red (blue). The gray area shows the time window of precision measurement, *T* − *t*_0_. The transition time *t*_0_ decreases from left to right and from top to bottom. The respective precision cost *F*_*P*_ and the *F*_1,*E*_- and *F*_1,*I*_-cost (without the factor *W*_1_) are given in the box of each figure.

Figure 12 shows optimal control signals and the resulting trajectories of the firing rates for several values of *T* and *t*_0_ for the DU1-task at point a for the highest possible value of *W*_1_, i.e., *W*_1,max_. We find two different control strategies. For large transition times, *t*_0_ ≳ *210ms, T*≳*230ms*, the optimal control remains a one-dimensional signal to the inhibitory population (see Figure 12, top row). Again, the cost remains almost constant with decreasing transition time, whereas the plateau state becomes shorter. For small transition times, *t*_0_ ≲ *200ms, T* ≲ *220ms*, the optimal control is a one-dimensional signal to the excitatory population (see Figure 12, bottom row). The amplitude increases only for *t*_0_ ≈ *0ms* and reaches a maximum of approximately 0.6 nA for *t*_0_ = *0ms*. *W*_1_ is a relatively high number, preventing large input signals at the cost of an increased precision cost *F*_*P*_.

Transition strategies differ from the solution found for *T* = *500ms* once the simulation duration becomes comparable to the width of the control signal, i.e., around *t*_0_ ≈ *180ms* and *T* ≈ *200ms*. For longer *t*_0_ and *T*, control signals are relatively similar to the original signal for *T* = *500ms* (see top panel in Figures 11, 12). For shorter *t*_0_ and *T*, the control signals differ notably from the original signal. The respective costs increase. For *t*_0_ ≲ *180ms* and *T* ≲ *200ms*, the results for $W_{1}=1\cdot \frac{1}{As^{5/2}}$ and *W*_1_ = *W*_1,max_ reveal different strategies. *W*_1_ determines the relationship between precision and control strength. In accordance with expectations, we can enforce either precise transitions, by choosing $W_{1}\approx 1\cdot \frac{1}{As^{5/2}}$, or low-amplitude transitions, by choosing $W_{1}\gg 1\cdot \frac{1}{As^{5/2}}$. For both penalization strategies, $W_{1}=1\cdot \frac{1}{As^{5/2}}$ and *W*_1_ = *W*_1,max_, it is more efficient to stimulate the inhibitory population for long transition times and the excitatory population for short transition times. This could be a consequence of the time delay *d*_*E*_ (see Equations 6 and 7) and of the fact that we measure precision only in the firing rate of the excitatory population, as *r*_*E*_ reacts faster to inputs to the excitatory node.

## 4. Discussion

This study uses an iterative numerical algorithm to compute optimal control for a biologically motivated nonlinear mean-field model of a population of excitatory and inhibitory neurons for four different control tasks. Our key findings are as follows: First, there are continuous sets of optimal control signals for each parameter choice and task if the time interval with no penalty on precision is sufficiently long, i.e., if the precision cost at the end of this interval is negligible compared to the cost of control strength. Since the duration of the control inputs remains finite even for long time intervals [0, *T*], time-shifted versions of otherwise identical control signals are cost-optimal as long as control signals are not too close to the interval boundaries. Second, we find that the optimal control operates such that the system is steered just minimally beyond the boundary that separates the two basins of attraction. The system converges to the respective stable target state without the requirement of further control input beyond that boundary. This keeps the control costs low. Third, we find systematic dependencies of input channels and certain parameters related to the shape of the optimal control signals on the distance to the target regime boundary. Rather unexpectedly, we also find that optimal control strategies do not consistently select one input channel, but steer the system through the excitatory or inhibitory channel depending on the exact location in state space. Finally, in a time-constrained setting, we observe not only amplitude effects, which would be expected, but also changes in shape and input channels.

Our approach to nonlinear OCT features some technical limitations, which must be considered appropriate to ensure that reliable results are produced. First, gradient descent algorithms are not guaranteed to converge to global optima. Optimal cost solutions may reflect local optima only, and there may be other initializations that could converge to control inputs at an even lower cost. Comparing solutions resulting from different initializations, however, did not provide evidence for a complicated energy landscape. One specific control signal shape is found from different initialization strategies, and shifts in time are computationally extremely time-consuming. We conclude that our heuristic approach to initialization produces results that are satisfactorily close to a global optimum and can thus be used to reliably investigate the systematic properties of optimal control strategies. Models of higher complexity, however, may require modifications of initialization strategies (cf. Chouzouris et al., 2021).

The time complexity of the proposed OCT method depends on the number of dynamical variables, the number of iterations of the descent algorithm, and the simulation time measured in units of the integration step size. Computation time scales linearly with the simulation time T and the number of iterations. The computation of the adjoint state (see Section 2.2.2, Figure 3, and Supplementary section 1) requires the Jacobian matrix. Hence, the computational complexity of the gradient of the cost scales quadratically with the number of dynamical variables. The computation of the descent step *s*_κ_ (see Section 2.2.2 and Figure 3) requires approximately O(10-1,000) forward simulations per descent step, the computational complexity of the forward simulation scales linearly with *N* and *T*. For our investigations, we find that due to a large number of forward simulations, the step size computation accounts for approximately 40–60% of the total computation time. For the EI EIF model, the computation of the optimal control signal for one initialization for one point in state space requires approximately 10 min CPU time on a laptop-computer (11th Gen Intel® Core™ i7-1165G7, CPU base frequency 2.8 GHz, maximum frequency 4.7 GHz) for *T* = *500ms* (integration step *dt* = *0.1ms*). The choice of abort criteria, $\epsilon_{s}=1\times 10^{-30}$ and $\epsilon_{\text{u}}=1\times 10^{-12}$ (see Section 2.2.2 and Figure 3), led to several thousand iterations of the gradient descent procedure. For simpler models (e.g., the Wilson-Cowan model), the computation time decreases approximately by a factor of *M*/*N*, where *M* is the number of the respective dynamical variables, rendering the investigation of neural mass models of complex networks feasible also on laptop computers (cf. Chouzouris et al., 2021).

Given the high metabolic demand of neural systems, evolutionary pressure could have enforced energy efficient interactions between its components (Niven, 2016; Watts et al., 2018). The consequences for the neural dynamics could, in principle, be investigated using methods from nonlinear OCT. Setting up a realistic energy balance for a neural system is a difficult task, and a neural mass model as it is investigated here would not be detailed enough to allow for this. Given the interpretation of the control **u**(*t*) as an induced ion current that affects the neurons' membrane potential, the metabolic energy *E* required to restore the neurons' state could be estimated roughly *via* the number of ions involved,^1^


(25) $$\begin{array}{l} E\propto \int_{0}^{T}\left(\left|u_{E}\left(t\right)|+|u_{I}\left(t\right)\right|\right)dt. \end{array}$$

In our simplistic example, efficiency would then be related to the *L*^1^-norm of the control, i.e., to the optima of the corresponding cost functional $F_{1}\left(\text{x},\text{u}\right)$ in Equation (19). The formalism of OCT investigated in this work, however, can be extended to other cost functionals in principle and may thus allow for realistic analytical investigations into the consequences of metabolic or other constraints on neural processing.

On the synthetic side, both the *L*^1^- and the *L*^2^-norm have previously been investigated in the context of the external control of neural systems. The *L*^2^-norm leads to so-called minimum-energy control strategies (cf. Nabi et al., 2012; Wilson et al., 2015). These strategies are motivated by reduced energy consumption of an electric stimulation device potentially supporting a longer-term deployment. The *L*^1^-norm leads to so-called minimum-charge control strategies (cf. Pyragas et al., 2018, 2020). These strategies are motivated by a reduced interference with neural tissue potentially lowering the danger of tissue damage (cf. Shannon, 1992). Gradient-based optimization as investigated in this study may provide an alternative method to derive these optimal control strategies. With properly adapted precision measures (e.g., measures of synchronization, Chouzouris et al., 2021) and alternative constraints (if required), the formalism of OCT investigated in this work can be extended to a variety of novel control goals.

This study focuses on a state-switching task in a bistable regime. *In vivo* experiments show that neural tissue can spontaneously transit between a state of low, steady activity (1 Hz-5 Hz) and a state of high activity or rhythmic bursting in the absence of stimuli (Latham et al., 2000; Holcman and Tsodyks, 2006). Electrophysiological recordings during the execution of memory tasks report regular transitions between states of inactivity and activity of single neurons (e.g., Funahashi et al., 1989). During sleep and anesthesia, slow-wave oscillations are observed and commonly modeled as periodic transitions of up and down states (Torao-Angosto et al., 2021). It is hypothesized that such transitions are fundamental for working memory and attention and for memory consolidation during sleep (Diekelmann and Born, 2010; Klinzing et al., 2019). Hence, bistability is thought to be a functionally important element of neural population dynamics, and efficient control of the population state may be a prerequisite for performing cognitive tasks (Durstewitz and Seamans, 2006). Beyond its biological importance, bistability enables stimulation that is limited in time and can yet produce sustainable changes in the activity of the system and is, therefore, a convenient dynamical regime for studies of control.

The results reported in this study pertain to the noise-free case. When additive noise affects the membrane currents μ_α_ (see Equation 5), the mean activities ${\bar{r}}_{E}$ and ${\bar{r}}_{I}$ of both excitatory and inhibitory populations decrease in the up state, and ${\bar{r}}_{I}$ increases in the down state. In addition, noise-induced transitions between up and down states may occur. The probability of spontaneous transitions increases with noise strength. The theoretical framework needs to be adapted by replacing the precision cost in Equation (11) with its expectation value. Practically, it is required to average over several noise realizations. Preliminary investigations into the optimal control for switching between the two stable states in the bistable regime show that both the amplitude *a*_α_ and the cost *c*_α_ of the control signals increase. As a result, the system is pushed closer to the target regime. The plateau state vanishes thus preventing immediate noise-induced transitions back to the original state.

In general, our theoretical and algorithmic approach can be applied to a wide range of models of neural dynamics, including whole-brain network structures (cf. Cakan et al., 2022) and can be extended to different control tasks (e.g., Chouzouris et al., 2021). This could, for example, open up new ways to study the efficiency of neural interaction theoretically. Evolutionary pressure and natural selection led to a high degree of cost efficiency in biological processes. Principles of communication resulting from applying optimal control to neural dynamics could thus be reflected in biological systems. In the context of our toy example, these principles could enable conclusions on the efficiency of stimulating the excitatory vs. the inhibitory population. On the synthetic side, applying optimal control methods to a real-world framework of neural dynamics could offer a fresh view on optimal protocols for neural stimulation in a clinical context, and presumably enable to minimize undesired side- and after-effects.

## Data availability statement

The data presented in this study can be found in the Github online repository: https://github.com/lenasal/Optimal_Control_GUI.

## Author contributions

LS implemented the algorithm, performed the simulations, and analyzed the data. KO supervised the project. Both authors conceptualized the study and drafted the manuscript.

## Funding

This work was supported by the DFG (German Research Foundation) *via* the CRC 910 (Project number 163436311).

## Conflict of interest

The authors declare that the research was conducted in the absence of any commercial or financial relationships that could be construed as a potential conflict of interest.

## Publisher's note

All claims expressed in this article are solely those of the authors and do not necessarily represent those of their affiliated organizations, or those of the publisher, the editors and the reviewers. Any product that may be evaluated in this article, or claim that may be made by its manufacturer, is not guaranteed or endorsed by the publisher.

## Footnotes

^1^For different ion species with different restoration costs the integrand must be replaced by a weighted sum of the individual currents.
