# Supplementary material for: Nonlinear optimal control of a mean-field model of neural population dynamics
Source: Front Comput Neurosci. 2022 Aug 3;16:931121. doi: 10.3389/fncom.2022.931121 (PMC9382303; doi:10.3389/fncom.2022.931121)
Supplement: Supplementary file 1 [file Data_Sheet_1.PDF]

Supplementary Material

1 STABILITY ANALYSIS AT EQUILIBRIUM STATES

We perform a stability analysis at different points $(\mu_E^{\mathrm{ext}}, \mu_I^{\mathrm{ext}})$ within the bistable regime. This requires the Jacobian matrix J of the system of ordinary differential equations (ODEs) that corresponds to our system of delay differential-algebraic equations (DDAEs) given in Equation (5), section 2.1.2 in the main article. The eigenvalues of J evaluated at an equilibrium state provide insights into the type of equilibrium: If all eigenvalues have negative real parts, the equilibrium is asymptotically stable. Else, it is unstable (Izhikevich (2007)).

To find an expression for the Jacobian matrix, we reformulate Equation (5), section 2.1.2 in the main article and split it into a set of differential and a set of algebraic equations. We collect the differential variables in the vector $\mathbf{y}$ and algebraic variables in the vector $\mathbf{z}$,

$$\mathbf{y} = \begin{pmatrix} \mu_E \\ \mu_I \\ \bar{s}_{EE} \\ \bar{s}_{EI} \\ \bar{s}_{IE} \\ \bar{s}_{II} \\ \sigma_{s,EE} \\ \sigma_{s,EI} \\ \sigma_{s,IE} \\ \sigma_{s,II} \end{pmatrix}, \mathbf{z} = \begin{pmatrix} r_E \\ r_I \\ \sigma_E \\ \sigma_I \\ \tau_E \\ \tau_I \end{pmatrix}. \tag{S1}$$

With this notation we can express the dynamics of the system as

$$\begin{aligned} \dot{\mathbf{y}} &= \mathbf{g}_1(\mathbf{y}(t), \mathbf{z}(t), \mathbf{z}(t-d_E), \mathbf{z}(t-d_I), \mathbf{u}(t)) \\ 0 &= \mathbf{g}_2(\mathbf{y}(t), \mathbf{z}(t), \mathbf{z}(t-d_E), \mathbf{z}(t-d_I)). \end{aligned} \tag{S2}$$

In our formulation (see Equation (18), section 2.2.3 in the main article), control only enters the differential equations, i.e., only g_1 is a function of $\mathbf{u}(t)$.

For the stability analysis, we only consider situations where $\mathbf{u} = 0$ and all dynamical variables $\mathbf{y}$ and $\mathbf{z}$ do not change over an extended period of time. Therefore, we set $d_E = d_I = 0$. Equation (S2) simplifies to

$$\begin{aligned} \dot{\mathbf{y}} &= \mathbf{g}_1(\mathbf{y}(t), \mathbf{z}(t)) \\ 0 &= \mathbf{g}_2(\mathbf{y}(t), \mathbf{z}(t)). \end{aligned} \tag{S3}$$

Following Riaza (2008), we find for $\dot{\mathbf{z}}$

$$\frac{d\mathbf{g}_2}{dt} = 0 = \frac{\partial \mathbf{g}_2}{\partial \mathbf{z}}\dot{\mathbf{z}} + \frac{\partial \mathbf{g}_2}{\partial \mathbf{y}}\dot{\mathbf{y}} \quad \Rightarrow \quad \dot{\mathbf{z}} = -\left(\frac{\partial \mathbf{g}_2}{\partial \mathbf{z}}\right)^{-1} \frac{\partial \mathbf{g}_2}{\partial \mathbf{y}} \underbrace{\mathbf{g}_1(\mathbf{y}, \mathbf{z})}_{\dot{\mathbf{y}}} := \mathbf{g}_3(\mathbf{y}, \mathbf{z}). \tag{S4}$$

We thus obtain a system of ODEs, which describes the dynamics of the mean-field EI EIF model,

$$\begin{aligned}\dot{\mathbf{y}} &= \mathbf{g}_1(\mathbf{y}, \mathbf{z}) \\ \dot{\mathbf{z}} &= \mathbf{g}_3(\mathbf{y}, \mathbf{z}).\end{aligned} \tag{S5}$$

The Jacobian matrix J of this system of ODEs,

$$J = \begin{pmatrix} \frac{\partial \mathbf{g_1}}{\partial \mathbf{y}} & \frac{\partial \mathbf{g_1}}{\partial \mathbf{z}} \\ \frac{\partial \mathbf{g_3}}{\partial \mathbf{y}} & \frac{\partial \mathbf{g_3}}{\partial \mathbf{z}} \end{pmatrix}, \tag{S6}$$

can be evaluated numerically.

We conduct simulations with a long duration ($T \geq 1000\,\mathrm{ms}$) and initialize the simulations such that the system converges to either the down or the up state. After approximately $10\,\mathrm{ms}$, the system reaches a stable state that does not change for the remaining time of the simulation. Numerical simulations hence strongly indicate stable equilibria. As a sanity check, we compute the eigenvalues of the Jacobian matrix evaluated at the stable states of low and high activity for various points in the bistable regime. We denote the final values of all dynamical variables by $\mathbf{x}_{\text{down}}$ and $\mathbf{x}_{\text{up}}$. We insert these values into the Jacobian matrix J (see Equation (S6)), find that all eigenvalues are non-positive, and validate that the equilibria are stable.

We observe a plateau state in all dynamical variables during optimal transitions (see section 3.2 in the main article). More precisely, there is a time interval $[t_{P_0}, t_{P_1}] \subseteq [250\,\mathrm{ms}, 400\,\mathrm{ms}]$, during which all components of $\mathbf{x}$ change only marginally. We denote this plateau state as $\mathbf{x}_P$ and hypothesize that it is an equilibrium state, as $\dot{\mathbf{x}}_P = 0$. For each point $(\mu_E^{\text{ext}}, \mu_I^{\text{ext}})$ in state space, the plateau state differs. The times t_{P_0} and t_{P_1} might vary not only with changing parameter combinations $(\mu_E^{\text{ext}}, \mu_I^{\text{ext}})$, but also with the control task. We select $[t_{P_0}, t_{P_1}]$ as the longest possible time interval within $[250\,\mathrm{ms}, 400\,\mathrm{ms}]$, such that both r_E and r_I change at most by $0.1\,\mathrm{Hz}$ over the whole interval. We compute the plateau state $\mathbf{x}_P$ by averaging over this interval,

$$\mathbf{x}_P = \frac{1}{t_{P_1} - t_{P_0}} \int_{t_{P_0}}^{t_{P_1}} \mathbf{x}(t)\; dt. \tag{S7}$$

We evaluate the Jacobian matrix at $\mathbf{x}_P$ for various points $(\mu_E^{\text{ext}}, \mu_I^{\text{ext}})$ in state-space, and calculate its eigenvalues. We find eigenvalues with positive real parts and validate that $\mathbf{x}_P$ is unstable.

2 OPTIMAL CONTROL OF A NONLINEAR SYSTEM OF DIFFERENTIAL ALGEBRAIC EQUATIONS WITH THE ADJOINT METHOD

Our model of neural dynamics is described by a system of DDAEs (see Equation (S3) and Equation (5), section 2.1.2 in the main article). We rephrase Equation (10), section 2.2.1 in the main article,

$$\begin{aligned}\mathcal{F}(\mathbf{z}(t), \tilde{\mathbf{z}}(t), \mathbf{u}(t)) = F_P + W_1 \cdot F_1 + W_2 \cdot F_2 &:= \int_0^T f(\mathbf{z}(t), \tilde{\mathbf{z}}(t), \mathbf{u}(t))\; dt \\ &= \int_0^T f_P(\mathbf{z}(t), \tilde{\mathbf{z}}(t)) + W_1 \cdot f_1(\mathbf{u}(t)) + W_2 \cdot f_2(\mathbf{u}(t))\; dt.\end{aligned} \tag{S8}$$

$\tilde{\mathbf{z}}(t)$ denotes the target state, which is measured in the algebraic variable only, and $\mathbf{u}(t)$ denotes the control. Time delays do not enter the cost functional. The optimal control minimizes the cost (see Equation (14),

section 2.2.1 in the main article),

$$\mathbf{u}^* = \arg\min_{\mathbf{u}} \mathcal{F}(\mathbf{z}(t), \tilde{\mathbf{z}}(t), \mathbf{u}(t)). \tag{S9}$$

We want to compute the gradient $\nabla_{\mathbf{u}}\mathcal{F}$ of the cost functional with respect to the control. To this end, we introduce the Hamiltonian (Biegler (2010))

$$\mathcal{H} = \int_0^T f(\mathbf{y}, \mathbf{z}, \tilde{\mathbf{z}}, \mathbf{u}) + \boldsymbol{\lambda}_1^T \cdot (\mathbf{g}_1(\mathbf{y}, \mathbf{z}, \mathbf{z}_E, \mathbf{z}_I, \mathbf{u}) - \dot{\mathbf{y}}) + \boldsymbol{\lambda}_2^T \cdot \mathbf{g}_2(\mathbf{y}, \mathbf{z}, \mathbf{z}_E, \mathbf{z}_I)\, dt, \tag{S10}$$

with g_1 and g_2 as given in Equation (S2). We introduce the short-hand notation $\mathbf{z}_\alpha := \mathbf{z}(t - d_\alpha)$, $\alpha \in E, I$. We use partial integration to replace the time derivative of $\mathbf{y}$,

$$\int_0^T \boldsymbol{\lambda}_1^T \cdot \dot{\mathbf{y}}\, dt = \left[\boldsymbol{\lambda}_1^T \cdot \mathbf{y}\right]_0^T - \int_0^T \dot{\boldsymbol{\lambda}}_1^T \cdot \mathbf{y}\, dt, \tag{S11}$$

and write the Hamiltonian as

$$\mathcal{H} = \int_0^T H\, dt - \left[\boldsymbol{\lambda}_1^T \cdot \mathbf{y}\right]_0^T, \tag{S12}$$

with

$$H = f(\mathbf{y}, \mathbf{z}, \tilde{\mathbf{z}}, \mathbf{u}) + \boldsymbol{\lambda}_1^T \cdot \mathbf{g}_1(\mathbf{y}, \mathbf{z}, \mathbf{z}_E, \mathbf{z}_I, \mathbf{u}) + \boldsymbol{\lambda}_2^T \cdot \mathbf{g_2}(\mathbf{y}, \mathbf{z}, \mathbf{z}_E, \mathbf{z}_I) + \dot{\boldsymbol{\lambda}}_1^T \cdot \mathbf{y}. \tag{S13}$$

$\boldsymbol{\lambda}_1(t)$ and $\boldsymbol{\lambda}_2(t)$ are Lagrange multipliers that have the same dimensions as the differential and algebraic variables respectively, and are referred to as the adjoint states. If the optimal control $\mathbf{u}^*(t)$ is applied, all partial derivatives of the Hamiltonian H vanish. We denote the corresponding states as $\mathbf{y}^*(t)$ and $\mathbf{z}^*(t)$.

In the following, we explicitly derive the gradient $\nabla_{\mathbf{u}}\mathcal{F}$ of the cost functional with respect to the control for the case without delay, i.e., $d_E = d_I = 0$. We then point out the changes when considering a system with time delay, i.e., $d_E, d_I \neq 0$. For a thorough derivation in the latter case, we refer to Göllmann et al. (2009).

In the case $d_E = d_I = 0$, we obtain the optimality conditions

$$\begin{aligned} \frac{\partial H}{\partial \mathbf{y}} &= \frac{\partial f}{\partial \mathbf{y}} + (\boldsymbol{\lambda}_1^*)^T \cdot \frac{\partial \mathbf{g_1}}{\partial \mathbf{y}} + (\boldsymbol{\lambda}_2^*)^T \cdot \frac{\partial \mathbf{g_2}}{\partial \mathbf{y}} + (\dot{\boldsymbol{\lambda}}_1^*)^T = 0 \quad \text{and} \\ \frac{\partial H}{\partial \mathbf{z}} &= \frac{\partial f}{\partial \mathbf{z}} + (\boldsymbol{\lambda}_1^*)^T \cdot \frac{\partial \mathbf{g_1}}{\partial \mathbf{z}} + (\boldsymbol{\lambda}_2^*)^T \cdot \frac{\partial \mathbf{g_2}}{\partial \mathbf{z}} = 0. \end{aligned} \tag{S14}$$

These two coupled differential equations define the adjoint states. The gradient of the cost functional (see Equation (S8)) with respect to the control can be written as

$$\begin{aligned}
\nabla_{\mathbf{u}}\mathcal{F} =& \nabla_{\mathbf{u}}\mathcal{H} = \int_0^T \nabla_{\mathbf{u}} H \, dt \\
=& \int_0^T \frac{\partial f}{\partial \mathbf{y}} \cdot \frac{\partial \mathbf{y}}{\partial \mathbf{u}} + \frac{\partial f}{\partial \mathbf{z}} \cdot \frac{\partial \mathbf{z}}{\partial \mathbf{u}} + \frac{\partial f}{\partial \mathbf{u}} + (\boldsymbol{\lambda}_1)^T \cdot \left(\frac{\partial \mathbf{g_1}}{\partial \mathbf{y}} \cdot \frac{\partial \mathbf{y}}{\partial \mathbf{u}} + \frac{\partial \mathbf{g}_1}{\partial \mathbf{z}} \cdot \frac{\partial \mathbf{z}}{\partial \mathbf{u}} + \frac{\partial \mathbf{g_1}}{\partial \mathbf{u}} \right) \\
&+ (\boldsymbol{\lambda}_2)^T \cdot \left(\frac{\partial \mathbf{g_2}}{\partial \mathbf{y}} \cdot \frac{\partial \mathbf{y}}{\partial \mathbf{u}} + \frac{\partial \mathbf{g}_2}{\partial \mathbf{z}} \cdot \frac{\partial \mathbf{z}}{\partial \mathbf{u}} + \frac{\partial \mathbf{g_2}}{\partial \mathbf{u}} \right) + \dot{\boldsymbol{\lambda}}_1^T \cdot \frac{\partial \mathbf{y}}{\partial \mathbf{u}} \, dt \\
=& \int_0^T \frac{\partial \mathbf{y}}{\partial \mathbf{u}} \cdot \left(\frac{\partial f}{\partial \mathbf{y}} + \boldsymbol{\lambda}_1^T \cdot \frac{\partial \mathbf{g}_1}{\partial \mathbf{y}} + \boldsymbol{\lambda}_2^T \cdot \frac{\partial \mathbf{g}_2}{\partial \mathbf{y}} + \dot{\boldsymbol{\lambda}}_1^T \right) + \frac{\partial \mathbf{z}}{\partial \mathbf{u}} \cdot \left(\frac{\partial f}{\partial \mathbf{z}} + \boldsymbol{\lambda}_1^T \cdot \frac{\partial \mathbf{g}_1}{\partial \mathbf{z}} + \boldsymbol{\lambda}_2^T \cdot \frac{\partial \mathbf{g}_2}{\partial \mathbf{z}} \right) \\
&+ \frac{\partial f}{\partial \mathbf{u}} + \boldsymbol{\lambda}_1^T \cdot \frac{\partial \mathbf{g}_1}{\partial \mathbf{u}} + \boldsymbol{\lambda}_2^T \cdot \frac{\partial \mathbf{g}_2}{\partial \mathbf{u}} \, dt
\end{aligned} \tag{S15}$$

The gradient simplifies when we insert the relations found in Equation (S14), as the terms in brackets vanish. We write $(\boldsymbol{\lambda}_1, \boldsymbol{\lambda}_2) = \boldsymbol{\lambda}$ and $(\mathbf{g}_1 - \dot{\mathbf{y}}, \mathbf{g}_2) = \mathbf{h}$, and denote the gradient and the Jacobian matrix with respect to the control by $\nabla_{\mathbf{u}}$ and $D_{\mathbf{u}}$, respectively. This leads to

$$\nabla_{\mathbf{u}}\mathcal{F} = \int_0^T \nabla_{\mathbf{u}} f + \boldsymbol{\lambda}^T \cdot D_{\mathbf{u}}\mathbf{h} \, dt. \tag{S16}$$

With $\mathbf{x} = (\mathbf{y}, \mathbf{z})$, we can also express the adjoint state equations (S14) as

$$\nabla_{\mathbf{x}} f_P + \boldsymbol{\lambda}^T \cdot D_{\mathbf{x}}\mathbf{h} - \dot{\boldsymbol{\lambda}}^T \cdot D_{\dot{\mathbf{x}}}\mathbf{h} = 0. \tag{S17}$$

$\nabla_{\mathbf{x}}$ is the gradient with respect to $\mathbf{x}$. $D_{\mathbf{x}}$ and $D_{\dot{\mathbf{x}}}$ are the Jacobian matrices with respect to the state variable and its derivative, respectively. Note that $\nabla_{\mathbf{x}} f = \nabla_{\mathbf{x}} f_P$ as $\nabla_{\mathbf{x}} f_{1,2} = 0$. Equation (S14) can be solved backwards in time with the final condition $\boldsymbol{\lambda}(T) = 0$.

With non-vanishing delays $d_E, d_I \neq 0$, the adjoint state equation (S17) reads

$$\nabla_{\mathbf{x}} f_P + \boldsymbol{\lambda}^T \cdot (D_{\mathbf{x}}\mathbf{h} + \chi_{[0,T-d_E]} D_{\mathbf{x}_E}\mathbf{h} + \chi_{[0,T-d_I]} D_{\mathbf{x}_I}\mathbf{h}) - \dot{\boldsymbol{\lambda}}^T \cdot D_{\dot{\mathbf{x}}}\mathbf{h} = 0. \tag{S18}$$

$\chi_{[t_a,t_b]}$ denotes the indicator function on the interval $[t_a, t_b]$. $D_{\mathbf{x}_E}$ and $D_{\mathbf{x}_I}$ are the Jacobian matrices with respect to the state variable at time $t - d_E$ (i.e., $\mathbf{x}(t - d_E)$) and at time $t - d_I$ (i.e., $\mathbf{x}(t - d_I)$). The expression for the gradient (see Equation (S16)) remains unchanged.

In both cases, the relation

$$\underbrace{\nabla_{\mathbf{u}} F_P}_{=0} + \sum_{j=1,2} W_j \cdot \nabla_{\mathbf{u}} F_j = \int_0^T \nabla_{\mathbf{u}} f \, dt \tag{S19}$$

must hold for f. This is satisfied with $\nabla_{\mathbf{u}} f = W_1 \cdot \nabla_{\mathbf{u}} f_1 + W_2 \cdot \nabla_{\mathbf{u}} f_2$, where (Casas et al. (2015))

$$(\nabla_{\mathbf{u}} f_1)_\alpha = \begin{cases} \frac{u_\alpha}{\sqrt{\int_0^T |u_\alpha|^2 \, dt}} \, dt & \text{if } \int_0^T |u_\alpha|^2 \neq 0 \\ 0 & \text{else} \end{cases}, \quad \alpha \in \{E, I\}, \tag{S20}$$
$$(\nabla_{\mathbf{u}} f_2)_\alpha = |u_\alpha|, \quad \alpha \in \{E, I\}.$$

3 VALIDATION OF RESULTS WITH SMALLER INTEGRATION STEP

To validate our results and prove their robustness towards numerical errors, we perform the optimization with a ten times smaller integration step ($dt = 0.01\,\mathrm{ms}$ instead of $dt = 0.1\,\mathrm{ms}$) for the DU1-task at point a with $W_1 = 1 \cdot \frac{1}{\mathrm{As}^{5/2}}$, for simulation times $T = 500\,\mathrm{ms}$, $T = 100\,\mathrm{ms}$, and $T = 25\,\mathrm{ms}$. Computation times with $dt = 0.01\,\mathrm{ms}$ are approximately 10 times longer than with $dt = 0.1\,\mathrm{ms}$.

We plot the the optimal control signal and population activity for $dt = 0.1\,\mathrm{ms}$ and $dt = 0.01\,\mathrm{ms}$ and the respective differences in Figure S1 for $T = 500\,\mathrm{ms}$. There are no qualitative differences in the control signal, it differs by $\lesssim 2\,\mathrm{pA}$. Activities are slightly shifted and differ by $\approx 3\,\mathrm{Hz}$. The resulting costs are comparable.

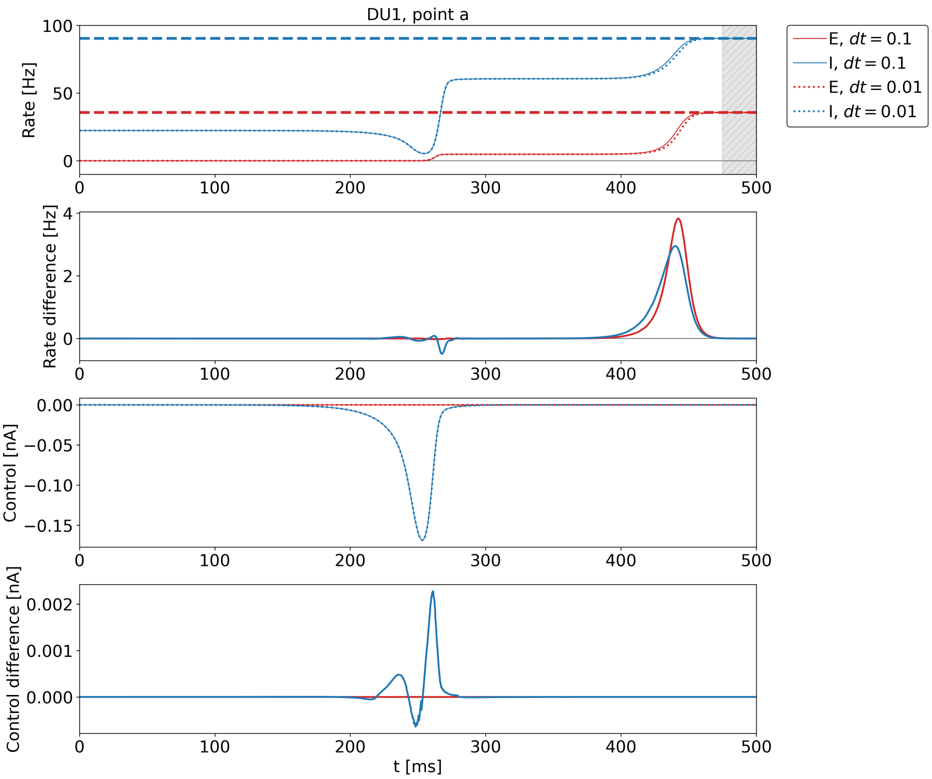

Figure S1. Optimal control signals and resulting firing rates for integration steps $dt = 0.1\,\mathrm{ms}$ (solid lines) and $dt = 0.01\,\mathrm{ms}$ (dotted lines) and their respective differences at point a for the DU1-task, with $T = 500\,\mathrm{ms}$ and $W_1 = 1 \cdot \frac{1}{\mathrm{As}^{5/2}}$. r_E and u_E are plotted in red color, r_I and u_I in blue color. The gray-shaded area marks the time interval of precision cost measurement. With $dt = 0.1\,\mathrm{ms}$, cost contributions are $F_P = 1.225 \times 10^{-5}$ and $F_1 = 3.331$. With $dt = 0.01\,\mathrm{ms}$, cost contributions are $F_P = 3.514 \times 10^{-5}$ and $F_1 = 3.341$.

Figures S2 and S3 show the equivalent plots for the simulation times $T = 100\,\mathrm{ms}$ and $T = 25\,\mathrm{ms}$. Control signals are very similar and differ by $\lesssim 2.5\,\mathrm{pA}$ ($T = 100\,\mathrm{ms}$) and $\lesssim 50\,\mathrm{pA}$ ($T = 25\,\mathrm{ms}$). The firing rates are again slightly shifted against each other and differ by $\approx 0.5\,\mathrm{Hz}$ ($T = 100\,\mathrm{ms}$) and by $\approx 6\,\mathrm{Hz}$ ($T = 25\,\mathrm{ms}$). The resulting costs are comparable.

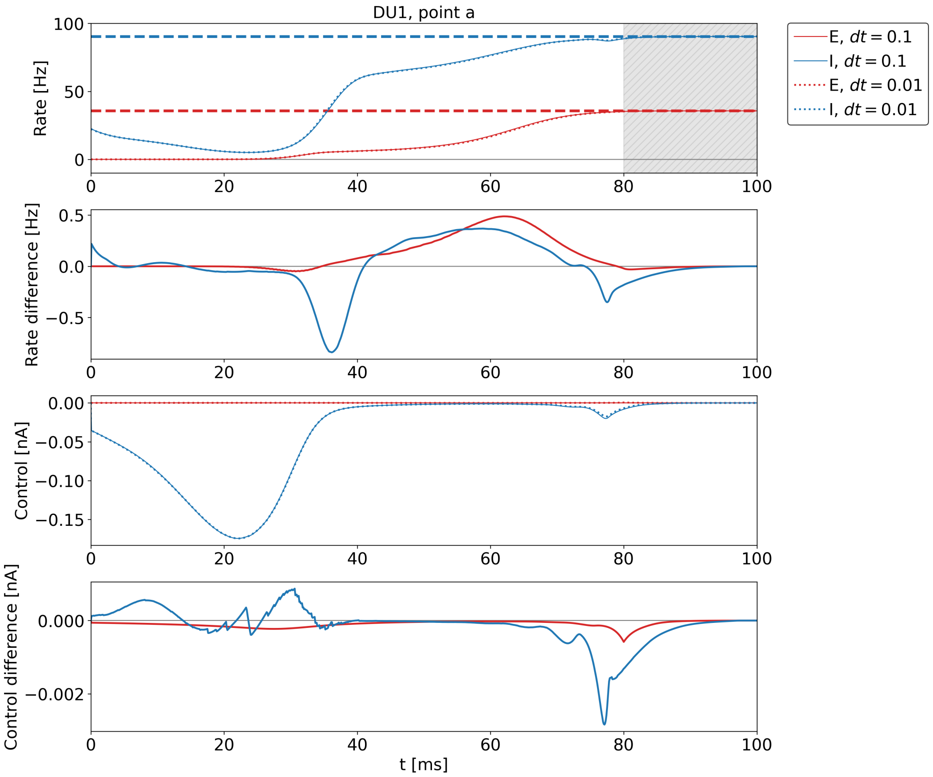

Figure S2. Optimal control signals and resulting firing rates for integration steps $dt = 0.1\,\mathrm{ms}$ (solid lines) and $dt = 0.01\,\mathrm{ms}$ (dotted lines) and their respective differences at point a for the DU1-task, with $T = 100\,\mathrm{ms}$ and $W_1 = 1 \cdot \frac{1}{\mathrm{As}^{5/2}}$. r_E and u_E are plotted in red color, r_I and u_I in blue color. The gray-shaded area marks the time interval of precision cost measurement. With $dt = 0.1\,\mathrm{ms}$, cost contributions are $F_P = 0.1201$ and $F_1 = 3.428$. With $dt = 0.01\,\mathrm{ms}$, cost contributions are $F_P = 0.09822$ and $F_1 = 3.437$.

From these comparisons, we conclude that the integration step of $dt = 0.1\,\mathrm{ms}$ is sufficient to produce qualitatively correct results.

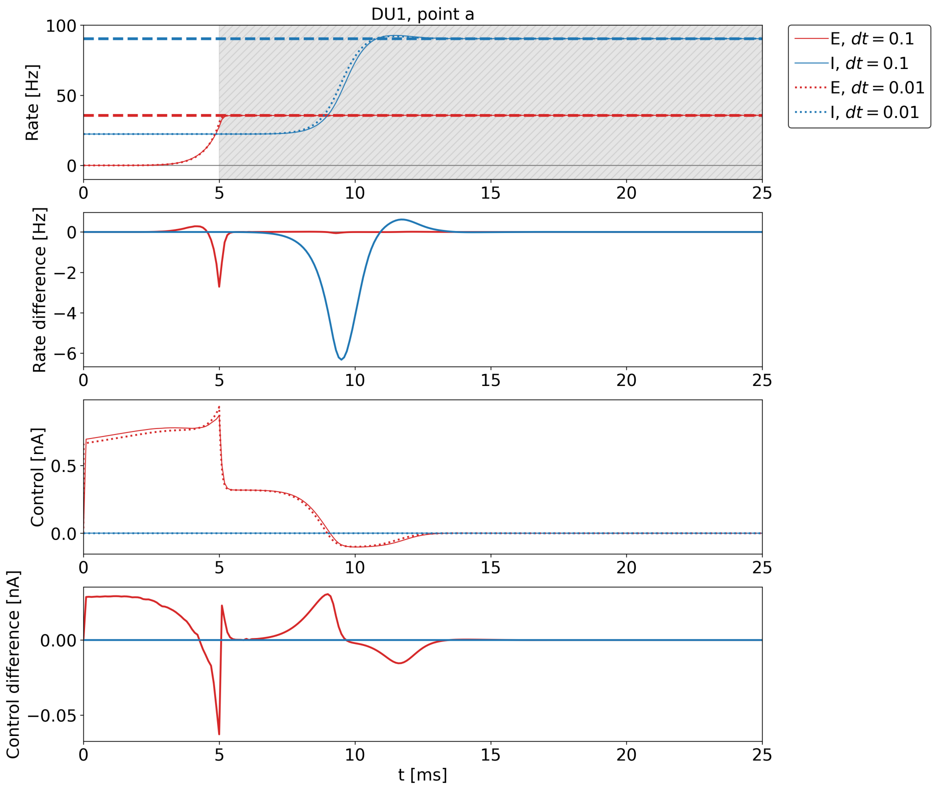

Figure S3. Optimal control signals and resulting firing rates for integration steps $dt = 0.1\,\mathrm{ms}$ (solid lines) and $dt = 0.01\,\mathrm{ms}$ (dotted lines) and their respective differences at point a for the DU1-task, with $T = 25\,\mathrm{ms}$ and $W_1 = 1 \cdot \frac{1}{\mathrm{As}^{5/2}}$. r_E and u_E are plotted in red color, r_I and u_I in blue color. The gray-shaded area marks the time interval of precision cost measurement. With $dt = 0.1\,\mathrm{ms}$, cost contributions are $F_P = 0.531$ and $F_1 = 9.018$. With $dt = 0.01\,\mathrm{ms}$, cost contributions are $F_P = 0.5336$ and $F_1 = 8.846$.

4 TRADEOFFS BETWEEN TRANSITION TIME AND COST

In section 3.4 in the main article, we show the optimal control signals for transitions with $T \leq 500\,\mathrm{ms}$ for the DU1-task at point a. For such transitions under time constraints, it is relevant whether we measure precision costs in r_E only, in both r_E and r_I, or in r_I only. Results for the first case are discussed in the main article, results for the second and third case are shown in Figures S4 and S5 respectively, for the DU1-task at point a, for $W_1 = 1 \cdot \frac{1}{\mathrm{As}^{5/2}}$.

If precision is measured in r_E and r_I, we observe three different control strategies. For long transition times, $t_0 \gtrsim 90\,\mathrm{ms}, T \gtrsim 110\,\mathrm{ms}$, the optimal control remains a one-dimensional signal to the inhibitory population (see Figure S4, top row). The cost remains almost constant with decreasing transition time, however, the plateau state becomes shorter. For intermediate transition times, $16\,\mathrm{ms} \lesssim t_0 \lesssim 80\,\mathrm{ms}, 36\,\mathrm{ms} \lesssim T \lesssim 100\,\mathrm{ms}$, there is a finite contribution of u_E that increases when t_0 becomes smaller, but input to the excitatory population is smaller than the input to the inhibitory population, i.e., $F_{1,E} < F_{1,I}$ (see Figure S4, center row). In both input channels u_E and u_I, a secondary peak appears just before t_0, which helps pushing the system towards the target state in both populations. For small transition times, $t_0 \lesssim 15\,\mathrm{ms}, T \lesssim 35\,\mathrm{ms}$, the input to the excitatory population is larger than the input to the inhibitory population, i.e., $F_{1,E} > F_{1,I}$ (see Figure S4, bottom row). The amplitudes increase and reach a maximum of approximately $a_E = 8\,\mathrm{nA}$ and $a_I = 5\,\mathrm{nA}$ for $t_0 = 0\,\mathrm{ms}$. With this control strength, the firing rates of both populations reach the target state after approximately $2\,\mathrm{ms}$.

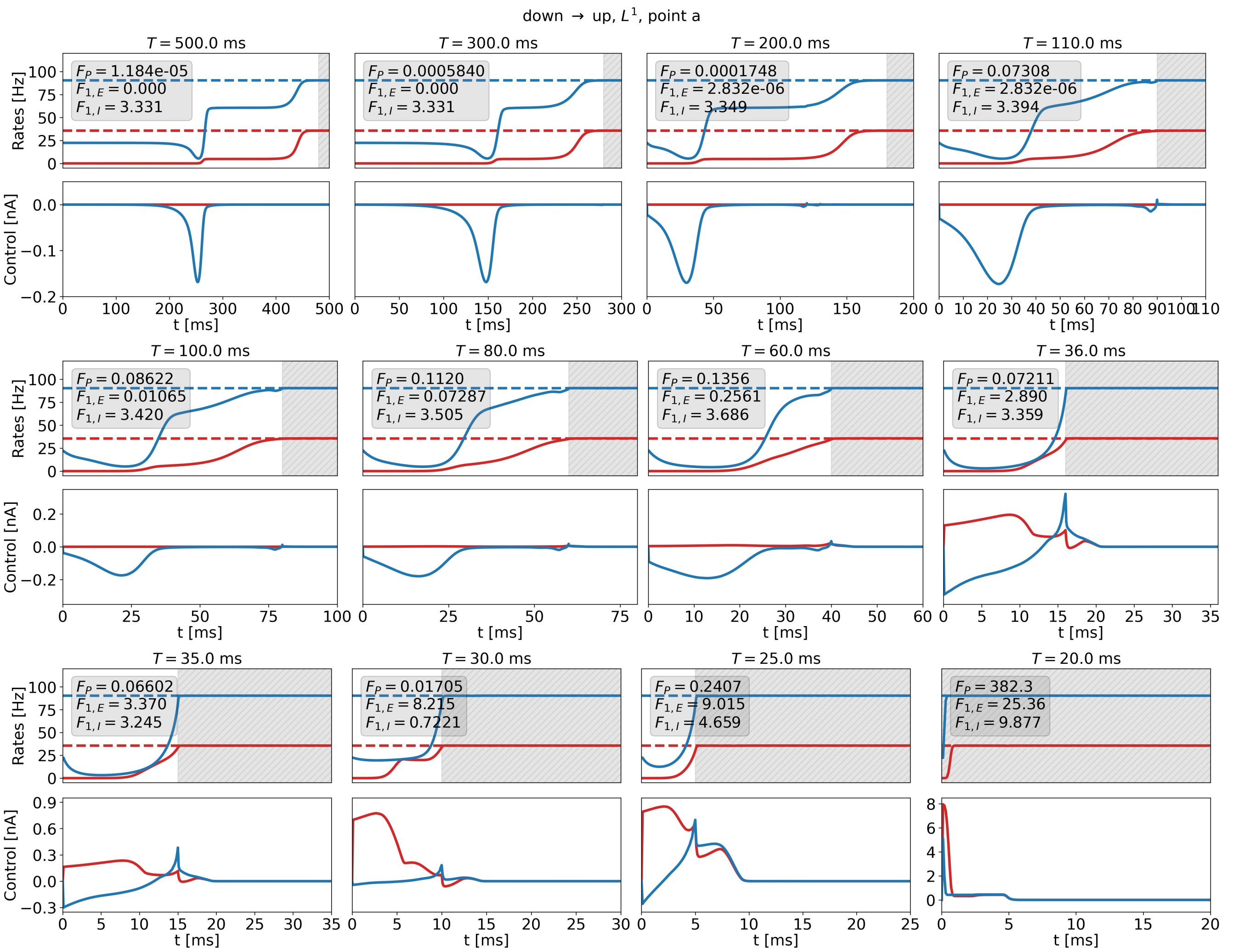

Figure S4. Firing rates (top panels) and optimal control signals (bottom panels) for transitions with various transition times t_0 for the DU1-task at point a for for $W_1 = 1 \cdot \frac{1}{\mathrm{As}^{5/2}}$, when precision is measured in the firing rates of both excitatory and inhibitory population. Excitatory (inhibitory) activity and control applied to the excitatory (inhibitory) population are plotted in red (blue). The gray area shows the time window of precision measurement. The transition time t_0 decreases from left to right and from top to bottom. The respective precision cost F_P, and the $F_{1,E}$- and $F_{1,I}$-cost are given in the box of each figure.

If precision is measured in r_I only, we observe three different control strategies. For long transition times $t_0, T \gg 0\,\mathrm{ms}$, the optimal control remains a one-dimensional signal to the inhibitory population (see Figure S5, top row). At first ($t_0 \gtrsim 180\,\mathrm{ms}, T \gtrsim 200\,\mathrm{ms}$), the cost remains almost constant with decreasing transition time, whereas the plateau state becomes shorter. A secondary peak appears just before t_0, which helps pushing the system towards the target state. For intermediate transition times, $9\,\mathrm{ms} \lesssim t_0 \lesssim 16\,\mathrm{ms}, 29\,\mathrm{ms} \lesssim T \lesssim 36\,\mathrm{ms}$, there is a finite contribution of u_E that increases and becomes comparable to u_I with decreasing t_0 becomes smaller (see Figure S4, center row). For small transition times, $t_0 \lesssim 8\,\mathrm{ms}, T \lesssim 28\,\mathrm{ms}$, the optimal control is a prolonged, constant signal to the inhibitory population during the precision measurement, i.e., in the time interval $[t_0, T]$ (see Figure S5, bottom row). The rate of the excitatory population remains in its low state, i.e., $r_E \approx 0\,\mathrm{Hz}$. With this signal, we can control the rate of the inhibitory population only, without affecting the rate of the excitatory population. A short peak

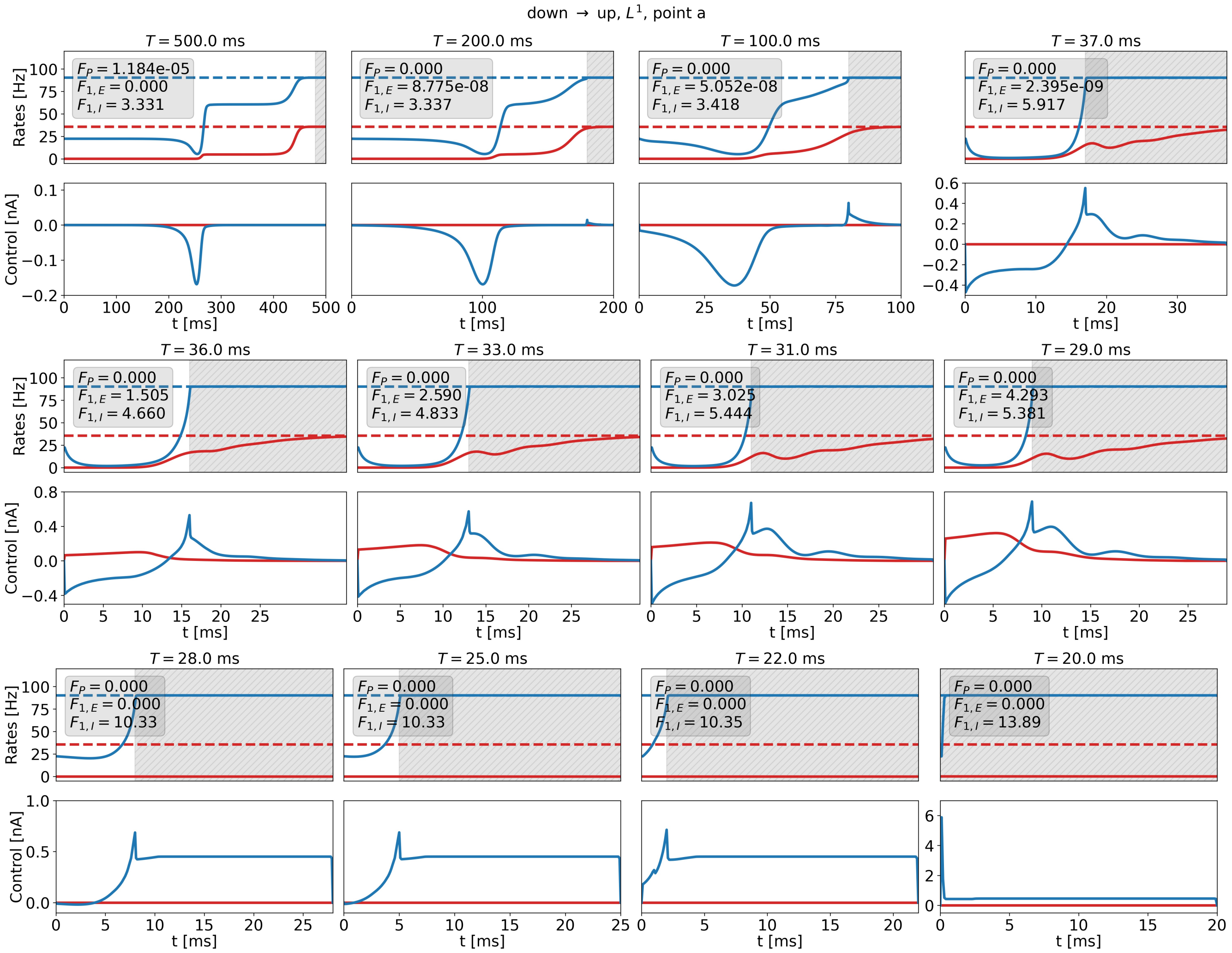

Figure S5. Firing rates (top panels) and optimal control signals (bottom panels) for transitions with various transition times t_0 for the DU1-task at point a for for $W_1 = 1 \cdot \frac{1}{\mathrm{As}^{5/2}}$, when precision is measured in the firing rate of the inhibitory population. Excitatory (inhibitory) activity and control applied to the excitatory (inhibitory) population are plotted in red (blue). The gray area shows the time window of precision measurement. The transition time t_0 decreases from left to right and from top to bottom. The respective precision cost F_P, and the $F_{1,E}$- and $F_{1,I}$-cost are given in the box of each figure.

before t_0 helps pushing r_I towards its target state, afterwards the control is required to maintain this value. The amplitude increases for $t_0 \approx 0\,\mathrm{ms}$ and reaches a maximum of approximately $6\,\mathrm{nA}$ for $t_0 = 0\,\mathrm{ms}$.

Comparing these results also with the results presented in section 3.4 in the main article, we validate that the transition strategies do not differ for long transition times $t_0 \gg 0\,\mathrm{ms}$. For short transition times, it is more efficient to stimulate the population(s) in which precision costs are measured in all cases. We hypothesize that this is a consequence of the time delays d_E and d_I (see Equations (6) and (7), section 2.1.2 in the main article). Control inputs to the population α affect the firing rate of the same population, r_α, faster than the firing rate of the opposite population. Hence, in a time-constraint setting, stimulating the population of precision measurement reduces precision costs.

REFERENCES

Biegler, L. (2010). *Nonlinear Programming: Concepts, Algorithms, and Applications to Chemical Processes*. MOS-SIAM Series on Optimization (Society for Industrial and Applied Mathematics)

Casas, E., Herzog, R., and Wachsmuth, G. (2015). Analysis of spatio-temporally sparse optimal control problems of semilinear parabolic equations. *ESAIM: Control, Optimisation and Calculus of Variations* 2015. doi:10.1051/cocv/2015048

Göllmann, L., Kern, D., and Maurer, H. (2009). Optimal control problems with delays in state and control variables subject to mixed control–state constraints. *Optimal Control Applications and Methods* 30, 341–365. doi:https://doi.org/10.1002/oca.843

Izhikevich, E. (2007). *Dynamical Systems in Neuroscience*. Computational neuroscience Dynamical systems in neuroscience (MIT Press)

Riaza, R. (2008). *Differential-Algebraic Systems* (WORLD SCIENTIFIC). doi:10.1142/6746
